# Activated neutrophils polarize protumorigenic interleukin‐17A‐producing T helper subsets through TNF‐α‐B7‐H2‐dependent pathway in human gastric cancer

**DOI:** 10.1002/ctm2.484

**Published:** 2021-06-27

**Authors:** Zhi‐guo Shan, Jun Chen, Jin‐shan Liu, Jin‐yu Zhang, Ting‐ting Wang, Yong‐sheng Teng, Fang‐yuan Mao, Ping Cheng, Quan‐ming Zou, Wei‐ying Zhou, Liu‐sheng Peng, Yong‐liang Zhao, Yuan Zhuang

**Affiliations:** ^1^ Department of General Surgery and Center of Minimal Invasive Gastrointestinal Surgery Southwest Hospital Third Military Medical University Chongqing China; ^2^ Department of General Surgery Qijiang Hospital of the First Affiliated Hospital of Chongqing Medical University Qijiang Chongqing China; ^3^ National Engineering Research Center of Immunological Products Department of Microbiology and Biochemical Pharmacy College of Pharmacy Third Military Medical University Chongqing China; ^4^ Chongqing Key Research Laboratory for Drug Metabolism Department of Pharmacology Chongqing Medical University Chongqing China; ^5^ Department of Gastroenterology the Affiliated Hospital of Southwest Medical University Luzhou Sichuan China; ^6^ Jiangsu Key Laboratory of Medical Science and Laboratory Medicine School of Medicine Jiangsu University Jiangsu China

**Keywords:** B7‐H2, gastric cancer, IL‐17A, neutrophils

## Abstract

**Rationale:**

Neutrophils constitute massive cellular constituents in inflammatory human gastric cancer (GC) tissues, but their roles in pathogenesis of inflammatory T helper (Th) subsets are still unknown.

**Methods:**

Flow cytometry analysis and immunohistochemistry were used to analyze the responses and phenotypes of neutrophils in different samples from 51 patients with GC. Kaplan‐Meier plots and Multivariate analysis for the survival of patients were used by log‐rank tests and Cox proportional hazards models. Neutrophils and CD4^+^ T cells were purified and cultured for *ex vivo*, *in vitro* and *in vivo* regulation and function assays.

**Results:**

GC patients exhibited increased tumoral neutrophil infiltration with GC progression and poor patient prognosis. Intratumoral neutrophils accumulated in GC tumors via CXCL6/CXCL8‐CXCR1‐mediated chemotaxis, and expressed activated molecule CD54 and co‐signaling molecule B7‐H2. Neutrophils induced by tumors strongly expressed CD54 and B7‐H2 in both dose‐ and time‐dependent manners, and a close correlation was obtained between the expressions of CD54 and B7‐H2 on intratumoral neutrophils. Tumor‐derived tumor necrosis factor‐α (TNF‐α) promoted neutrophil activation and neutrophil B7‐H2 expression through ERK‐NF‐κB pathway, and a significant correlation was found between the levels of TNF‐α and CD54^+^ or B7‐H2^+^ neutrophils in tumor tissues. Tumor‐infiltrating and tumor‐conditioned neutrophils effectively induced IL‐17A‐producing Th subset polarization through a B7‐H2‐dependent manner ex vivo and these polarized IL‐17A‐producing Th cells exerted protumorigenic roles by promoting GC tumor cell proliferation via inflammatory molecule IL‐17A in vitro, which promoted the progression of human GC in vivo; these effects could be reversed when IL‐17A is blocked. Moreover, increased B7‐H2^+^ neutrophils and IL‐17A in tumors were closely related to advanced GC progression and predicted poor patient survival.

**Conclusion:**

We illuminate novel underlying mechanisms that TNF‐α‐activated neutrophils link B7‐H2 to protumorigenic IL‐17A‐producing Th subset polarization in human GC. Blocking this pathological TNF‐α‐B7‐H2‐IL‐17A pathway may be useful therapeutic strategies for treating GC.

## Graphical abstract

Our results illuminate a novel mechanism that TNF‐α‐activated neutrophils link B7‐H2 to protumorigenic IL‐17A‐producing Th subset polarization in human GC. Blocking this pathological TNF‐α‐B7‐H2‐IL‐17A pathway may be useful therapeutic strategies for treating GC.

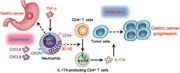

**Abbreviations**
ERK: extracellular signal‐regulated kinase
GC: gastric cancer
IL: interleukin
NF‐κB: nuclear factor κB
NTCS: non‐tumor tissue culture supernatants
PBMCs: peripheral blood mononuclear cells
Th: T helper
TNF‐α: tumor necrosis factor‐α
TTCS: tumor tissue culture supernatants

## 1 INTRODUCTION

Gastric cancer (GC), as one of the most common tumors, is regarded as the second cause of cancer‐related death in the world.^1^ Presently, GC pathogenesis is still not identified, but the development of GC is closely bound up with the cross‐talks among various immune cells in GC tumors.^2^, ^3^ Increasing evidence suggests that the proinflammatory responses induced by innate and adaptive immune cells in GC tumors can be rerouted into a tumor‐promoting direction.^4^, ^5^ A variety of immune cells infiltrate in GC tumors, and neutrophils and T cells, as the most abundant innate or adaptive immune cells respectively, are mostly infiltrated immune cells with profound immunoregulatory effects on tumor progression.^6^

Neutrophils markedly outnumber other leukocytes, once activated, they can express multiple surface molecules that provide specifically targeted responses to malignancy.^7^, ^8^ It has been shown that elevated peripheral neutrophils^9^ or intratumoral neutrophils^10^ predict poor GC patient survival, suggesting that neutrophils mostly have pathological promoting roles on GC. Inflammatory T helper (Th) subsets (also called CD4^+^ T cells) represent major populations of T cells in many types of cancers,^11^ and their differentiation is regulated by the networks comprising both surface and secretory factors.^12^, ^13^, ^14^ We have shown that interleukin (IL)‐17A‐producing Th subsets were highly enriched in GC, and their levels were closely associated with GC progression.^15^ Presently, there are so many areas unknown about the regulatory mechanism and potential role of these IL‐17A‐producing Th subsets in GC tumor immunopathology. Thus, to explore the polarization and underlying function of IL‐17A‐producing Th subsets in immunopathogenesis, there is a need to study the regulatory mechanism of IL‐17A‐producing Th subsets under inflamed conditions. Since neutrophils are the leukocytes which most frequently interact with T cells,^6^, ^16^, ^17^ it is important to determine whether and, if so, in GC tumors, how neutrophils orchestrate regulatory and functional processes of IL‐17A‐producing Th subsets.

Here, we investigate the interaction among neutrophils, CD4^+^ T cells, and tumor cells in tumor tissues, and find that, in GC, neutrophils can be recruited to GC tumors via CXCL6/CXCL8‐CXCR1‐mediated chemotaxis. Furthermore, tumor‐derived TNF‐α promotes neutrophil activation and neutrophil B7‐H2 expression by the activation of pathways of extracellular signal‐regulated kinase (ERK) and the activation and translocation of phosphorylated transcription factor nuclear factor κB (NF‐κB) into nucleus. Finally, these neutrophils induce the polarization of IL‐17A‐producing Th subsets in a B7‐H2‐dependent manner, which can increase the proliferation of GC cells *in vitro* and promote the progression and growth of human GC *in vivo* through inflammatory molecule IL‐17A. Collectively, our data illuminate pathogenic roles of neutrophils in GC with a novel mechanism that TNF‐α‐activated neutrophils link B7‐H2 to protumorigenic IL‐17A‐producing Th subset polarization in human GC milieu. The increase of these tumor‐infiltrating B7‐H2‐expressing neutrophils predicts GC progression and bad prognosis, suggesting that these cells and their regulating downstream networks may be served as novel targets in the therapy of GC.

## 2 MATERIALS AND METHODS

### 2.1 Patients and samples

Tumor tissues, peritumoral tissues (3‐5 cm from the edges of tumor tissues) and non‐tumor tissues (normal gastric tissues) (no less than 5 cm from the edges of tumor tissues), and the peripheral blood of autologous patients with GC were collected. These patients with GC did not receive any chemotherapy or radiation and then underwent surgical resection at Qijiang Hospital or Southwest Hospital. Patients with diseases of autoimmune and infection, or with other primary malignant tumor were eliminated. The clinical scale of GC was rated in accordance with the TNM classification criterion of the International Union Against Cancer (the eighth version). Antibodies and reagents used in this research are shown in Supplemental Table 1. Also, the main clinic characteristics of the patients with GC in this research are shown in Supplemental Table 2.

### 2.2 Tissues single‐cell isolation

According to the established methods,^6^, ^18^ the tissue samples were first washed two to four times with PBS, and then were cut into shreds. Then these tissues were put into RPMI 1640 medium containing fetal calf serum (FCS) (5%), deoxyribonuclease I (10 mg/ml), and collagenase IV (1 mg/ml), and were then mechanically separated in MACS Dissociator. Furthermore, the cell suspensions of dissociated tissues were enzymatically dispersed at 37°C for 1 h. After that, the cell suspensions of dissociated tissues were sieved using the filters (70 µm, BD Labware). The trypan blue exclusion staining was performed to determine the cell viability (> 95%).

### 2.3 Neutrophil and CD4^+^ T cell isolation

According to the established methods,^6^, ^18^ the single cells from tissues were prepared as above, and was then labeled with anti‐CD45, anti‐CD11b, anti‐CD66b, and anti‐CD15 antibodies. And autologous tumor or non‐tumor neutrophils from patients with GC were acquired using fluorescence‐activated cell sorting (FACS) (FACSAria II) on the gate of CD45^+^CD11b^+^CD66b^+^CD15^+^ live cells. The peripheral blood mononuclear cells (PBMCs) of autologous patients with GC or healthy adult donors were then obtained using Ficoll‐Paque Plus to perform centrifuging with density gradients. Peripheral neutrophils were ultimately collected by further red blood cell lysing using lysing solution. CD4^+^ T cells were sorted with anti‐CD4 (StemCell Technologies) magnetic beads from the previously isolated PBMCs. Finally, all of the sorted cells were used in this study only when the purity and viability were all determined > 95% (As for the contaminant cells, we think they may be apoptotic neutrophils).^6^, ^18^

**HIGHLIGHTS**

- GC tumor‐derived TNF‐α activates neutrophils and induces neutrophil B7‐H2 expression via ERK‐NF‐κB signaling pathway
- Tumor‐associated neutrophils induce IL‐17A‐producing Th subset polarization through a B7‐H2‐dependent manner, which promotes GC tumor cell proliferation via IL‐17A and contributes to human GC progression
- Increased B7‐H2^+^ neutrophils and IL‐17A in GC correlate with advanced tumor progression and predict poor patient survival

### 2.4 Tumor tissue culture supernatants (TTCS) or non‐tumor tissue culture supernatants (NTCS) and supernatant‐conditioned neutrophils preparation

According to the established methods,^6^, ^18^ TTCS and NTCS were acquired by placing autologous tumor tissues or non‐tumor tissues from patients with GC (with equal mass and from equal total recovered protein) in RPMI 1640 medium (1 ml) for 24 h. Then the supernatants were harvested by further centrifugation. For supernatant‐conditioned neutrophils preparation, the neutrophils collected as above were incubated with autologous 50% TTCS or 50% NTCS from patients with GC for 12 h, and next were cleaned three times using RPMI 1640 medium. The controls were prepared by culturing the neutrophils with RPMI‐1640 medium only.

### 2.5 Chemotaxis assay

According to the established methods,^6^, ^18^ we first added FACS‐sorted intratumoral neutrophils (1 × 10^5^) from tumor tissues of GC patients into the upper chambers of Transwells (3‐µm pore size). Then we added autologous 50% TTCS or NTCS into the lower chambers of Transwells (3‐µm pore size) to perform the chemotaxis assay. After 30‐min culture, migration of intratumoral neutrophils was then quantified by counting the neutrophils in the lower chamber. In other cases, before chemotaxis assay, we added 20 µg/ml CXCR1 blocking antibody (IgG2a) or 20 µg/ml control IgG2a into the suspensions of intratumoral neutrophils, then incubated for 2 h. In some other assays, we also added 20 µg/ml CXCL6 and/or CXCL8 blocking antibody (IgG1) or 20 µg/ml control IgG1 into the TTCS. Furthermore, human recombinant (hr) CXCL6 and/or CXCL8 (100 ng/ml) or RPMI‐1640 medium was placed in the lower chambers of Transwells (3‐µm pore size) as positive or normal control respectively.

### 2.6 Neutrophil stimulation

According to the established methods,^6^, ^18^ neutrophils from healthy donors (2 ml RPMI 1640 medium, 2 × 10^6^ cells/well) were stimulated with autologous 50% TTCS or 50% NTCS from patients with GC for 12 h, or with 50% TTCS for 3, 6, or 12 h, or with TTCS (10%, 20%, 50%) for 12 h, or with 50% TTCS together with TNF‐α blocking antibody (20 µg/ml) for 12 h, or with 50% NTCS together with hr TNF‐α (100 ng/ml) for 12 h, or with other different hr cytokines (100 ng/ml) for 12 h. Then, the neutrophils were collected after stimulation for western blot analysis or flow cytometric analysis. As for the inhibiting experiments of signaling pathways, the neutrophils were pre‐incubated with GSK‐3β inhibitor, AG490, U0126, FLLL32, SB203580, BAY 11–7082, Wortmannin, or SP600125 (5 µl, 10 µM) for 1 h, then were incubated with hr TNF‐α (100 ng/ml) or 50% TTCS for 12 h. The control parallel neutrophils were pre‐treated with culture media or dimethyl sulfoxide (DMSO) (5 µl).

### 2.7 Ex vivo and in vitro co‐culture of neutrophil and CD4^+^ T cells

According to the established methods,^6^, ^18^ in an *ex vivo* mixed culture system, we first labeled magnetic bead‐sorted peripheral CD4^+^ T cells (96‐well plates, 2 × 10^5^ cells/well) with carboxyfluorescein succinimidyl ester (CFSE), and then we mixedly cultured these CD4^+^ T cells with autologous tumor tissue or non‐tumor tissue neutrophils isolated from patients with GC at a 2:1 ratio (T cell : neutrophil)^5^, ^18^ in RPMI‐1640 medium (200 µl) (contained 5–10% FCS, 2 µg/ml anti‐CD3 antibodies, 1 µg/ml anti‐CD28 antibodies, and 20 IU/ml hr IL‐2), in the presence or absence of 20 µg/ml B7‐H2 blocking antibody (IgG2b) or 20 µg/ml control IgG2b. In an *in vitro* co‐culture system, we first labeled magnetic bead‐sorted peripheral CD4^+^ T cells (96‐well plates, 2 × 10^5^ cells/well) with CFSE, and then we mixedly cultured these CD4^+^ T cells with TTCS‐conditioned or NTCS‐conditioned neutrophils at a 2:1 ratio as described above, with or without 20 µg/ml B7‐H2 blocking antibody (IgG2b) or 20 µg/ml control IgG2b, or with or without 20 µg/ml CD54 blocking antibody (IgG1) or 20 µg/ml control IgG1. In transwell experiments, we first labeled magnetic bead‐sorted peripheral CD4^+^ T cells with CFSE, and we then transferred these CD4^+^ T cells in the lower chamber of Transwells (3‐µm pore size) in RPMI‐1640 medium (contained 5–10% FCS, 2 µg/ml anti‐CD3 antibodies, 1 µg/ml anti‐CD28 antibodies, and 20 IU/ml hr IL‐2), and we further added autologous TTCS‐conditioned neutrophils or autologous tumor‐infiltrating neutrophils either into the lower or the upper chamber of Transwells (3‐µm pore size) at a 2:1 ratio (T cell : neutrophil). As for the experiments of signaling pathway inhibition, we first labeled magnetic bead‐sorted peripheral CD4^+^ T cells (96‐well plates, 2 × 10^5^ cells/well) with CFSE, and we then pre‐incubated these CD4^+^ T cells with U0126 (10 µM) for 1 h, then mixedly cultured CD4^+^ T cells with autologous TTCS‐conditioned neutrophils or autologous tumor‐infiltrating neutrophils at a 2:1 ratio (T cell:neutrophil) in RPMI‐1640 medium (200 µl) (contained 5–10% FCS, 2 µg/ml anti‐CD3 antibodies, 1 µg/ml anti‐CD28 antibodies, and 20 IU/ml hr IL‐2). After 4‐day co‐culture, the cells were harvested for flow cytometric analysis, and the cell culture supernatants were collected for ELISA. In other cases, we first pretreated autologous tumor tissue or non‐tumor tissue neutrophils isolated from patients with GC, and TTCS‐ or NTCS‐conditioned neutrophils with or without 20 µg/ml B7‐H2 blocking antibody (IgG2b) or 20 µg/ml control IgG2b for 2 h. Then we labeled magnetic bead‐sorted peripheral CD4^+^ T cells (96‐well plates, 2 × 10^5^ cells/well) with CFSE, and we then co‐cultured these CD4^+^ T cells with those neutrophils above at a 2:1 ratio (T cell : neutrophil) in RPMI‐1640 medium (200 µl) (contained 5–10% FCS, 2 µg/ml anti‐CD3 antibodies, 1 µg/ml anti‐CD28 antibodies, and 20 IU/ml hr IL‐2). After 4‐day co‐culture, CD4^+^ T cells were further purified and collected for western blot analysis.

### 2.8 GC cell proliferation assay

According to the established methods,^6^, ^18^ SGC‐7901 was obtained from China Center for Type Culture Collection (CCTCC, China, verified by using short tandem repeat profiling methods), and was then stimulated with the culture supernatants from autologous peripheral CD4^+^ T cells and tumor‐associated neutrophils (a 4‐day *ex vivo* co‐culture system) with or without 20 µg/ml IL‐17A blocking antibody or 20 µg/ml control IgG, or the culture supernatants from autologous peripheral CD4^+^ T cells and TTCS‐conditioned neutrophils (a 4‐day *in vitro* co‐culture system) with or without 20 µg/ml IL‐17A blocking antibody or 20 µg/ml control IgG, or 100 ng/ml hr IL‐17A for 72 h. The GC cells were collected and the proliferation of GC cells was examined by CCK‐8 Kits (Dojindo)^18^ according to the instructions. Briefly, SGC‐7901 cells (96‑well plates, 1 × 10^4^ cells/well) were stimulated with the culture supernatants or IL‐17A as above for 72 h (with 3 replicates). The GC cell number was determined using the SpectraMax® i3x microplate reader (Molecular Devices) to examine the absorbance (450 nm). For Ki‐67 staining of GC cells, the GC cells were collected and then re‐suspended in cold ethanol (70%) and incubated for 1 h (‑20˚C). Then, the GC cells were stained with anti‑Ki‐67 antibody, and then were detected with FACSCanto™ (BD Biosciences).

### 2.9 In vivo GC tumor inhibition experiments

According to the established methods,^6^, ^18^ we collected SGC‐7901 cells (1 × 10^6^ cells in 100 µl of normal saline) then injected into the axillary subcutaneous of female nonobese diabetic/severe combined immunodeficiency (NOD/SCID) mice (6‐8 week, unilateral tumor‐burdened). Then we cultured neutrophils from peripheral blood of healthy adult donors with autologous 50% TTCS or 50% NTCS for 12 h. Then, we cultured autologous CD4^+^ T cells (5 × 10^6^ cells) mixedly with NTCS‐conditioned neutrophils (NCN) or TTCS‐conditioned neutrophils (TCN) at a 2:1 ratio (T cell:neutrophil) in RPMI‐1640 medium (contained 5–10% FCS, 2 µg/ml anti‐CD3 antibodies, 1 µg/ml anti‐CD28 antibodies, and 20 IU/ml hr IL‐2) for 4 days, and then we injected these cells (in 200 µl of normal saline) into the peritoneum on day 7 after inoculation. Furthermore, we injected IL‐17A blocking antibody or control IgG (in 100 µl of normal saline, 20 µg per mouse) into the peritoneum every 2 days after CD4^+^ T cell injection. In another experiment, we injected GC cells into the axillary tissues of the NOD/SCID mice as described above. And then we cultured neutrophils from peripheral blood of healthy adult donors with 50% TTCS for 12 h. Then, we co‐cultured autologous CD4^+^ T cells (5 × 10^6^ cells) with TTCS‐conditioned neutrophils (TCN) at a 2:1 ratio (T cell:neutrophil) in RPMI‐1640 medium (contained 5–10% FCS, 2 µg/ml anti‐CD3 antibodies, 1 µg/ml anti‐CD28 antibodies, and 20 IU/ml hr IL‐2), with or without 20 µg/ml B7‐H2 blocking antibody (IgG2b) or 20 µg/ml control IgG2b for 4 days, and we further injected these cells (in 200 µl of normal saline) into the peritoneum on day 7 after inoculated tumor. The measurement of tumorous size was done by two independent observers using vernier caliper every 3 days after inoculation. GC tumor volumes were calculated with the formula as below: volume = (B^2^ × A)/2 (B:rotational diameter; A:axial diameter). Finally, we sacrificed these mice, and photographed and further weighed GC tumors, then paraformaldehyde‐fixed GC tumors for further immunohistochemical staining.

### 2.10 Flow cytometric analysis

According to the established methods,^6^, ^18^ for surface molecule staining, the cells were labeled with specific antibodies or isotype antibodies. In cytokine staining, these cells were treated for 5 h with ionomycin (1 µg/ml) and phorbol myristate acetate (50 ng/ml) with GolgiStop. The cells were further fixed and permeated using Perm/Wash solution and then stained with specific antibodies. The samples were detected with FACSCanto™ (BD Biosciences), and the statistical analyses were performed with FACSDiva (BD Biosciences) or Flowjo software (TreeStar).

### 2.11 Western blot analysis

According to the established methods,^6^, ^18^ western blot examination were done on 8‐10% SDS‐PAGE gels using the same amount of proteins. PVDF membranes were blocked by using 5% BSA. We first detected human proteins of ERK1/2 and phosphorylated ERK1/2 (p‐ERK1/2), and p65 and phosphorylated p65 (p‐p65) with their specific antibodies respectively. Then we further incubated these proteins with the corresponding secondary antibodies that were conjugated with horseradish peroxidase (HRP) respectively. The amount of protein was visualized by SuperSignal® West Dura Extended Duration Substrate kit.

### 2.12 Enzyme‐linked immunosorbent assay

According to the established methods,^6^, ^18^ the cell culture supernatants were harvested as above for ELISA. The IL‐17A concentration in the neutrophil‐T cell co‐culture supernatants, the CXCL6, CXCL8, or TNF‐α concentration in TTCS or NTCS were measured by using the corresponding ELISA kits, respectively. We first harvested the autologous tumor and normal tissues and homogenized them in sterile Protein Extraction Reagent (1 ml). Then the tissue supernatants were used to analyze for the production of IL‐17A, CXCL6, CXCL8, and TNF‐α by using the corresponding ELISA kits, respectively. The total proteins were measured by using BCA Protein Assay Kit. The cytokine or chemokine concentrations in the tissues were expressed as picograms per milligrams of total protein.

### 2.13 Immunohistochemical staining

According to the established methods,^6^, ^18^ the samples were paraformaldehyde‐fixed and further paraffin‐embedded. Then the paraffin‐embedded tissue samples were sliced up into 5 µm slices. In immunohistochemical single‐staining, we incubated these slices with anti‐CD15, anti‐CD66b, anti‐proliferating cell nuclear antigen (PCNA), and anti‐IL‐17A antibodies, respectively, and we further incubated them with the corresponding anti‐mouse IgG/anti‐rabbit IgG which was conjugated with horseradish peroxidase (HRP) respectively, and then followed by diaminobenzidine. In immunohistochemical double‐staining, we incubated these slices with anti‐CD15 and anti‐CD66b antibodies, with anti‐CD15 and anti‐CD4 antibodies, or with anti‐CD4 and anti‐IL‐17A antibodies respectively, according to the instruction of ImmPRESS* Deut Double Staining Polymer Kit (anti‐mouse IgG/AP; anti‐rabbit IgG/HRP). Finally, the sections were counter‐stained with hematoxylin, and then were analyzed by using the microscope (Nikon Eclipse 80i; Nikon).

### 2.14 Immunofluorescence staining

According to the established methods,^6^, ^18^ we firstly blocked paraformaldehyde‐fixed tumor tissue sections from GC patients with 20% goat serum, and we then stained them for CD15 and CD4. Finally, the sections were analyzed by using the confocal fluorescence microscope (LSM 510 META, Zeiss).

### 2.15 Microarray analysis

The tumor tissues from patients with GC (10 GC patients) were collected, and Affymetrix GeneChip Human Gene 1.0 ST Array (Affymetrix) was used for analysis of the profiles of gene expression according to the manufacturer's protocol. Then the microarray wwas performed at the Genminix Informatics (China) with the microarray service certified by Affymetrix.

### 2.16 Real‐time PCR analysis

In accordance with the established methods,^6^, ^18^ we extracted total RNA from specimens, and we then reverse‐transcribed them into cDNA using PrimeScript™ Real‐time reagent Kit. We further used the Real‐time PCR Master Mix to perform real‐time PCR analysis on the IQ5 (Bio‐Rad). By using corresponding primers, we detected the IL‐17A, CXCL6, CXCL8, or TNF‐α expression in autologous tumor and normal tissues from patients with GC through the SYBR green method (Supplementary Table 3). The levels of human GAPDH mRNA were acted as normalizers, and the levels of gene expression in normal tissues were acted as calibrators. The relative expressions of genes were shown as the fold changes of relevant mRNA calculated by the ΔΔCt method, and the average levels of gene expression in non‐tumor tissues were defined as 1.

### 2.17 Statistical analysis

In this study, all the results are presented as mean±SEM. For the differences between two groups, student *t* test was mainly used, and when the variances differed, then the Mann‐Whitney U test was used. Pearson correlation analysis and linear regression analysis were used to analyze the correlations between parameters. Overall survival was standardized as the period between date of surgery and death. Deaths not related to surgery (eg, accidental death) were not considered final record for the study. Cumulative survival time was calculated using KM statistical method, and survival time was measured monthly; the log‐rank test was used to compare the differences between the two groups. When exploring the influencing factors that affect the survival of patients the Cox proportional hazards model was used. All statistical analyses were performed by SPSS 13.0 software. The data were kept to two decimal places, and *P* < 0.05 was regarded as the standard as statistically significant.

### 2.18 Study approval

The study was approved by the Ethics Committee of Qijiang Hospital and Southwest Hospital. These patients and healthy donors participating in the experiment have obtained informed consent. Breeding and experimentation of all animals were reviewed and approved by the Animal Ethical and Experimental Committee of Third Military Medical University.

## 3 RESULTS

### 3.1 Neutrophils are riched in tumor tissues as GC progress and indicate poor prognosis

**FIGURE 1:**
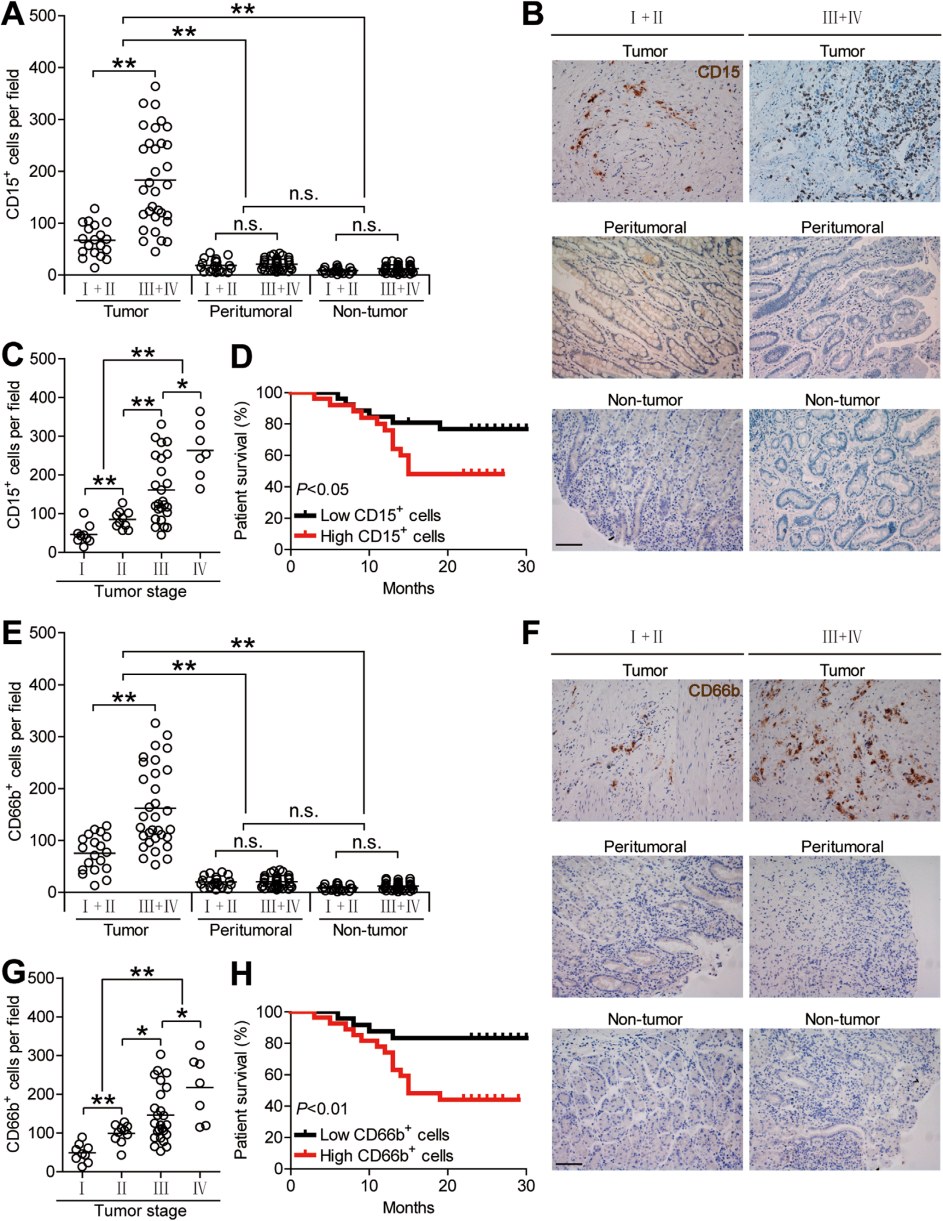
Neutrophils are increased in GC tumors as tumor progress and predict poor patient survival. (A) CD15^+^ neutrophil number among TNM stages (I+II vs III+IV) in each tissue of patients with GC by immunohistochemical staining and counting. Cumulative results from 51 GC patients were shown. (B) Representative analysis of CD15^+^ (brown) neutrophil distributions in tumor, peritumoral and non‐tumor tissues of GC patients by immunohistochemical staining. Scale bars: 100 microns. (C) Intratumoral CD15^+^ neutrophil number among TNM stages was compared. (D) Kaplan‐Meier plots for overall survival by median CD15^+^ neutrophil number (112 per field). (E) CD66b^+^ neutrophil number among TNM stages (I+II vs III+IV) in each tissue of patients with GC by immunohistochemical staining and counting. Cumulative results from 51 GC patients were shown. (F) Representative analysis of CD66b^+^ (brown) neutrophil distributions in tumor, peritumoral, and non‐tumor tissues of GC patients by immunohistochemical staining. Scale bars: 100 microns. (G) Intratumoral CD66b^+^ neutrophil number among TNM stages was compared. (H) Kaplan‐Meier plots for overall survival by median CD66b^+^ neutrophil number (111 per field). The horizontal bars in panels (A, C, E, or G) represent mean values. Each ring in panels (A, C, E, or G) represents one patient. *P < 0.05; **P < 0.01, ^n.s.^ *P *> 0.05 for groups connected by horizontal lines

To first evaluate the levels of neutrophils in GC tissues, we examined CD15^+^ neutrophil numbers in tumoral tissues, peritumoral tissues, or non‐tumor tissues of patients with GC with various stages, and we found that GC patients demonstrated higher CD15^+^ neutrophil numbers in tumoral tissues than those in peritumoral tissues and/or non‐tumor tissues (Figure 1A and B). Furthermore, we found that, with the progress of cancer, the numbers of tumoral CD15^+^ neutrophils increased significantly (Figure 1A), and this tumoral CD15^+^ neutrophil accumulation was noteworthy from stage I onward (Figure 1C). We further found that increased CD15^+^ neutrophil numbers were closely associated with increased tumor size as well as advanced tumor stage (Figure S1A). We next analyzed the clinical relevance of tumoral neutrophils in human GC. Comparing patients with high (≥112 median level) versus low (< 112) CD15^+^ neutrophil numbers, the 30‐month overall survival rates were significantly lower for those within the higher neutrophil number group (Figure 1D). Furthermore, the lower overall survival rates of 30 months for higher CD15^+^ neutrophil numbers were more pronounced in the GC patients with TNM stage (III+IV) (Figure S2A). Similar results were also obtained when we analyzed the number of CD66b^+^ neutrophils (Figure 1E‐H; Figures S1B and S2B). Furthermore, significant correlation was found between the CD15^+^ neutrophil numbers and the CD66b^+^ neutrophil numbers in GC tumors analyzed (Figure S3A), and co‐expression of CD15 and CD66b was found on the majority of neutrophils in tumors analyzed (Figure S3B). Altogether, these data suggest that tumoral neutrophils are increased and closely correlated with GC progression and poor prognosis of patients with GC.

### 3.2 Increased tumoral neutrophil accumulation is mediated by CXCL6/CXCL8‐CXCR1 chemotaxis axis

**FIGURE 2:**
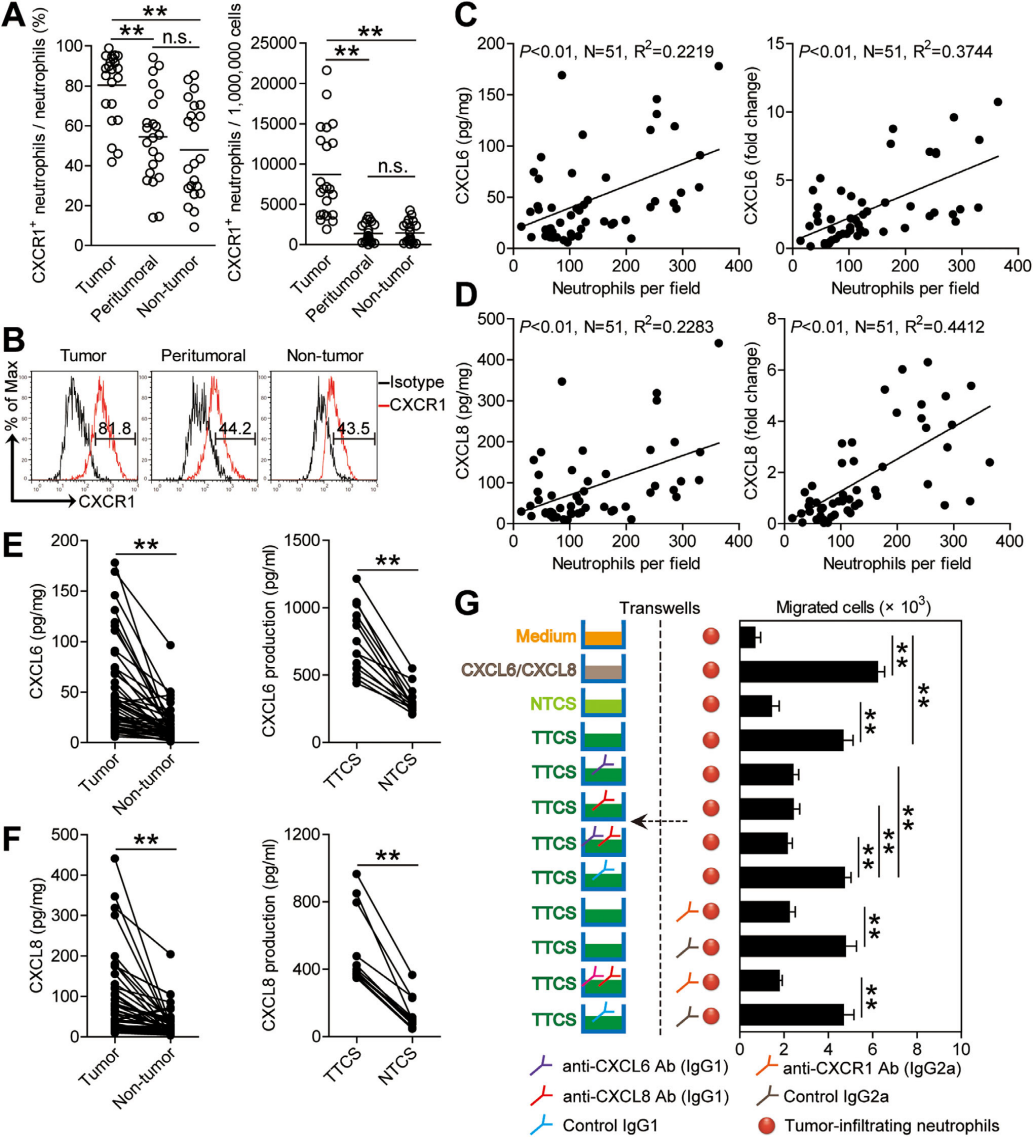
Increased neutrophil accumulation in GC tumors is promoted by CXCL6/CXCL8‐CXCR1‐mediated chemotaxis. (A) Statistics analysis of CXCR1^+^ neutrophil percentage in total neutrophils and the number of CXCR1^+^ neutrophils per million total cells in each samples of patients with GC by gating on CD45^+^CD11b^+^CD66b^+^CD15^+^CXCR1^+^ cells and counting (n = 22). (B) Expression of molecule CXCR1 on neutrophils. Color histograms represent staining of CXCR1; black, isotype control. (C) The correlations between neutrophils and CXCL6 in GC tumors were analyzed (n = 51). Results were expressed as neutrophil number and CXCL6 concentration/expression in tumor tissues. (D) The correlations between neutrophils and CXCL8 in GC tumors were analyzed (n = 51). Results were expressed as neutrophil number and CXCL8 concentration/expression in tumor tissues. (E) CXCL6 concentration between autologous tumor and non‐tumor tissues (n = 51) or between autologous TTCS and NTCS (n = 14) was analyzed. (F) CXCL8 concentration between autologous tumor and non‐tumor tissues (n = 51) or between autologous TTCS and NTCS (n = 14) was analyzed. (G) Migration of tumor‐infiltrating neutrophils was assessed by Transwell assay as described in Methods and statistically analyzed (n = 5). The horizontal bars in panels A represent mean values. Each ring or dot in panels (A, C, D, E, or F) represents one patient. Ab: antibody. *P < 0.05; **P < 0.01; ^n.s.^ *P *> 0.05 for groups connected by horizontal lines

The findings above suggest that tumor environments may trigger increased tumoral neutrophil accumulation, thus we hypothesized that tumor environments might promote the migration of neutrophils into GC tumor tissues through chemotaxis. So, the expression of the chemokine receptors on neutrophils in tumoral tissues as well as in peritumoral tissues or non‐tumor tissues was examined, and we found that tumoral neutrophils expressed significantly higher CXCR1 (Figure 2A and B) that has been reported to be involved in the chemotaxis of neutrophils,^19^, ^20^ while, neutrophils in peritumoral tissues or non‐tumor tissues expressed much lower CXCR1 (Figure 2A and B). However, there were no differences in the expressions of CCR1, CCR2, and CCR5 on neutrophils derived from different tissue locations (Figure S4A). Altogether, these findings manifest that the migration of neutrophils to tumor tissue is likely to be achieved through the chemokine CXCR1 pathway.

Furthermore, we next found that neutrophils were positively correlated with CXCL6 (Figure 2C) and CXCL8 (Figure 2D), the ligands for CXCR1, in GC tumors. Meanwhile, we found that the concentrations of CXCL6 (Figure 2E) and CXCL8 (Figure 2F) in tumor tissue culture supernatants (TTCS) or tumor tissues were obviously augmented in contrast with those in non‐tumor tissue culture supernatants (NTCS) or non‐tumor tissues. Similar findings were obtained when analyzing the expressions of CXCL6 and CXCL8 in tumor or non‐tumor tissues (Figure S4B). To further analyze the effects of CXCL6/CXCL8‐CXCR1 axis on neutrophil recruitment, tumor‐infiltrating neutrophils were isolated and neutrophil chemotaxis analysis was performed. The results showed that TTCS was superior to NTSC in inducing the migration of tumor‐infiltrating neutrophils, and this effect was almost lost when these neutrophils were pre‐treated with CXCL6/CXCL8 and/or CXCR1 blocking antibodies (Figure 2G). Altogether, these findings support a regulatory model wherein tumor tissues can produce CXCL6/CXCL8 that then recruits neutrophils into the GC environments mediated by CXCL6/CXCL8‐CXCR1 chemotaxis axis.

### 3.3 GC environments activate and induce neutrophil B7‐H2 expression

**FIGURE 3:**
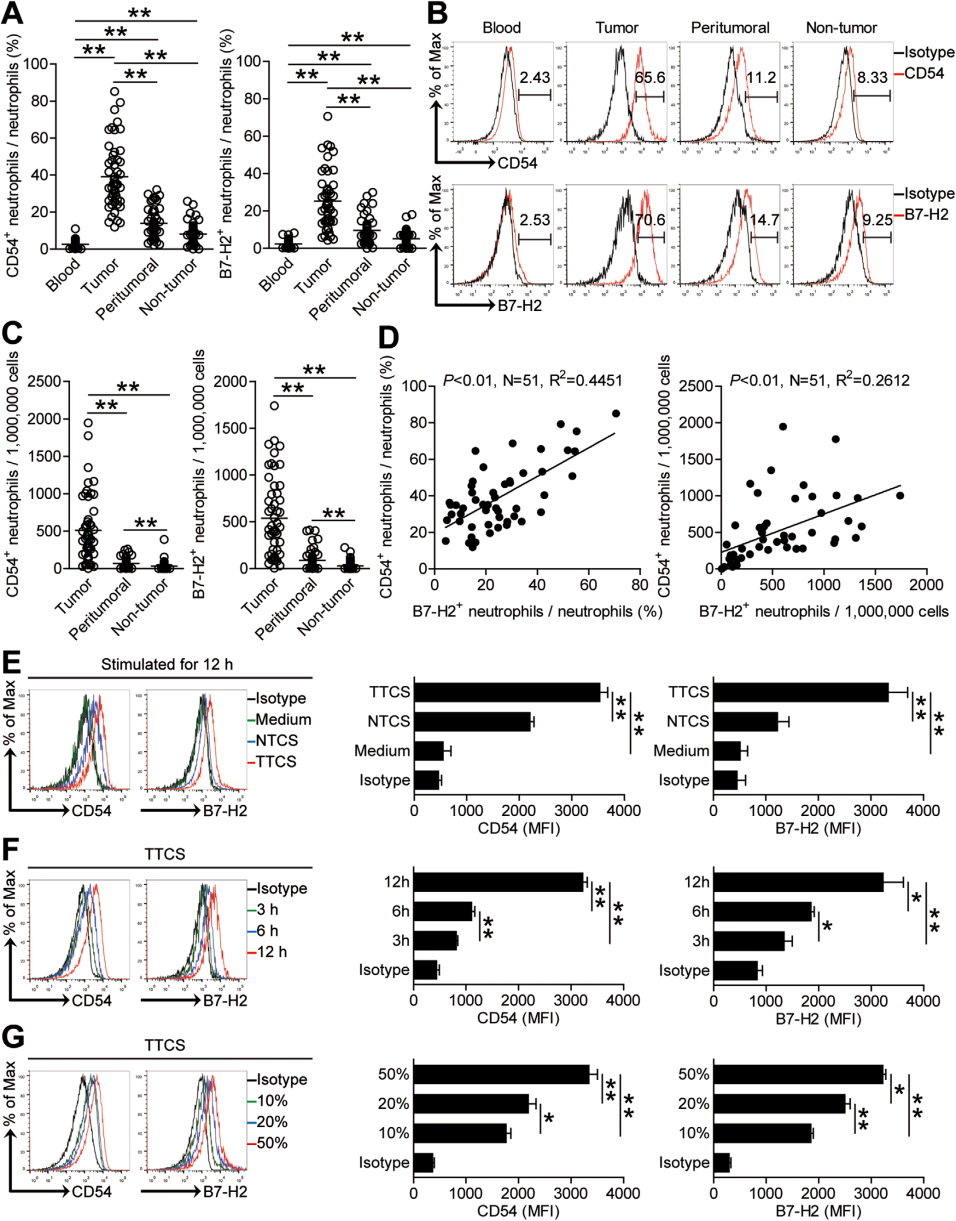
Human GC environments induce neutrophil activation and B7‐H2 expression. (A and C) Statistics analysis of CD54^+^ neutrophil percentage and B7‐H2^+^ neutrophil percentage in total neutrophils (A) and the number of CD54^+^ neutrophils and B7‐H2^+^ neutrophils per million total cells (C) in each samples of patients with GC by gating on CD45^+^CD11b^+^CD66b^+^CD15^+^CD54^+^ cells and CD45^+^CD11b^+^CD66b^+^CD15^+^B7‐H2^+^ cells and counting (n = 51). (B) Expression of molecule CD54 and B7‐H2 on neutrophils. Color histograms represent staining of CD54 and B7‐H2; black, isotype control. (D) The correlations between CD54^+^ neutrophils and B7‐H2^+^ neutrophils in human tumors were analyzed. Results are expressed as the percentage of CD54^+^ neutrophils and B7‐H2^+^ neutrophils in total neutrophils or the number of CD54^+^ neutrophils and B7‐H2^+^ neutrophils per million total cells in tumor tissues. (E) Representative data and statistical analysis of the expression of CD54 and B7‐H2 on neutrophils of healthy donors exposed to 50% autologous TTCS and NTCS for 12 hours (n = 3). black, isotype control. (F) Representative data and statistical analysis of the expression of CD54 and B7‐H2 on neutrophils exposed to 50% TTCS for 3, 6, 12 h (n = 3). black, isotype control. (G) Representative data and statistical analysis of the expression of CD54 and B7‐H2 on neutrophils exposed to 10%, 20%, 50% TTCS for 12 h (n = 3). black, isotype control. The horizontal bars in panels A or C represent mean values. Each ring or dot in panels (A, C, or D) represents one patient. MFI: mean fluorescence intensity. *P < 0.05; **P < 0.01 for groups connected by horizontal lines

Furthermore, we detected that tumoral neutrophils expressed manifestly higher percentage of CD54, an activation marker of neutrophils, than that expressed on neutrophils in peritumoral tissues or non‐tumor tissues (Figure 3A and B). Interestingly, tumoral neutrophils also expressed significantly higher percentage of co‐signaling molecule B7‐H2 than that expressed on neutrophils in peritumoral tissues or non‐tumor tissues (Figure 3A and B). However, blood neutrophils expressed little CD54 or B7‐H2 (Figure 3A and B). Similar findings were obtained when we analyzed the CD54^+^ neutrophil number or the B7‐H2^+^ neutrophil number in these tissues (Figure 3C). Furthermore, there were closely significant correlations between the expressing levels of CD54 as well as B7‐H2 on tumoral neutrophils (Figure 3D), indicating an activated B7‐H2‐expressing phenotype of tumoral neutrophils.

Meanwhile, we presumed that the GC tumors might lead to this activated B7‐H2‐expressing phenotype of tumoral neutrophils. Thus, we then stimulated blood neutrophils with autologous NTCS or TTCS from tumor tissues or non‐tumor tissues of patients with GC. Interestingly, TTCS‐conditioned neutrophils were superior to NTCS‐conditioned neutrophils in up‐regulating the expression of CD54 and B7‐H2 (Figure 3E). Furthermore, TTCS‐conditioned neutrophils could significantly up‐regulate the expressions of CD54 as well as B7‐H2 in both time‐ (Figure 3F) and dose‐dependent manners (Figure 3G). Altogether, the data above suggest that GC environments induce neutrophil activation and B7‐H2 expression.

### 3.4 Tumor‐derived TNF‐α promotes neutrophil activation and neutrophil B7‐H2 expression through ERK‐NF‐κB signaling pathway

**FIGURE 4:**
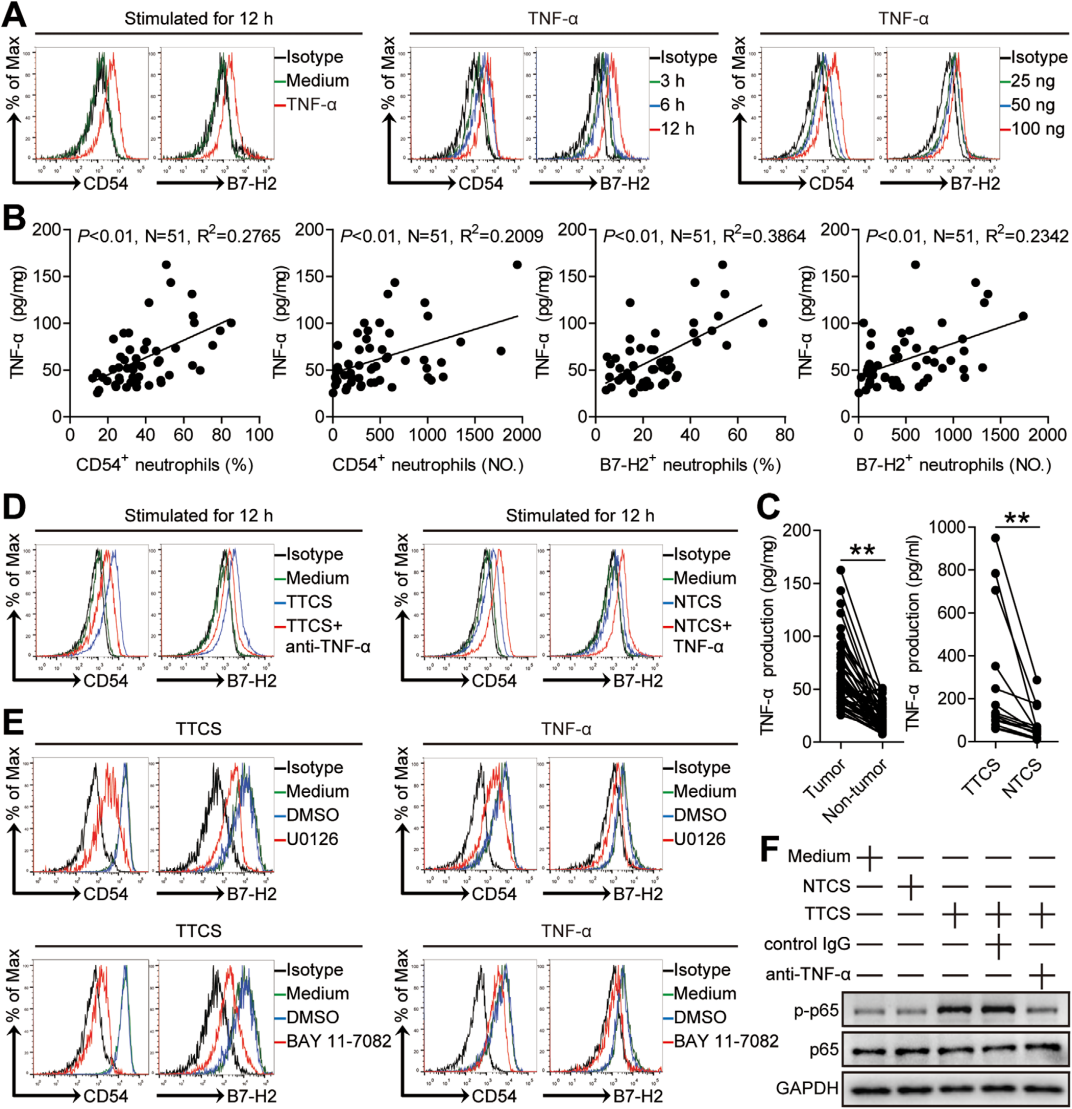
Tumor‐derived TNF‐α activates neutrophils and induces B7‐H2 expression on neutrophils via ERK‐NF‐κB pathway. (A) Expression of CD54 and B7‐H2 on neutrophils exposed to TNF‐α (100 ng/ml) or medium control for 12 h, or exposed to TNF‐α (100 ng/ml) for 3, 6, 12 h, or exposed to TNF‐α (25, 50, or 100 ng/ml) for 12 h. black, isotype control. (B) The correlations between TNF‐α and CD54^+^ neutrophils or B7‐H2^+^ neutrophils in human tumors were analyzed. Results are expressed as the percentage of CD54^+^ neutrophils and B7‐H2^+^ neutrophils in total neutrophils or the number of CD54^+^ neutrophils and B7‐H2^+^ neutrophils per million total cells and TNF‐α concentration in tumor tissues. (C) TNF‐α concentration between autologous tumor and non‐tumor tissues (n = 51) or between autologous TTCS and NTCS (n = 14) was analyzed. (D) Expression of CD54 and B7‐H2 on neutrophils exposed to TTCS with anti‐TNF‐α antibody or NTCS with TNF‐α for 12 h. (E) Expression of CD54 and B7‐H2 on neutrophils exposed to TTCS or TNF‐α with or without U0126 (an ERK inhibitor) or BAY 11‐7082 (an IκBα inhibitor) for 12 h. black, isotype control. (F) The p65 and p‐p65 proteins in neutrophils exposed to autologous TTCS, NTCS, or TTCS with anti‐TNF‐α antibody or control IgG for 12 h were analyzed by western blot. Each dot in panel (B) or (C) represents one patient. **P < 0.01 for groups connected by horizontal lines

Tumor environments can often produce different soluble proinflammatory cytokines. To see which proinflammatory cytokines promoted neutrophil B7‐H2 expression in GC, we first used microarray to screen the proinflammatory cytokines in tumor tissues (Figure S5A). Then we stimulated blood neutrophils with the highly‐expressed cytokines including granulocyte‐macrophage colony‐stimulating factor (GM‐CSF), macrophage colony‐stimulating factor (M‐CSF), granulocyte colony‐stimulating factor (G‐CSF), IL‐1β, IL‐4, IL‐6, IL‐10, IL‐12, IL‐17A, IL‐17F, IL‐21, IL‐23, IL‐33, TGF‐β, and TNF‐α. Interestingly, only TNF‐α could significantly up‐regulate neutrophil B7‐H2 expression (Figure 4A; Figure S5B). Furthermore, TNF‐α upregulated the expressions of CD54 and B7‐H2 on blood neutrophils in both time‐ and dose‐dependent means (Figure 4A). Moreover, the concentrations of TNF‐α were closely significant correlated with the levels of CD54^+^ neutrophils or B7‐H2^+^ neutrophils in tumor tissues (Figure 4B). And similar findings were obtained when we analyzed correlations between the expression of TNF‐α and the levels of CD54^+^ neutrophils or B7‐H2^+^ neutrophils in tumor tissues (Figure S6A). Moreover, TNF‐α concentration in TTCS or tumor tissues were significantly increased when compared to those in NTCS or non‐tumor tissues (Figure 4C). And similar findings were obtained when we analyzed TNF‐α expression between tumor tissues and non‐tumor tissues (Figure S6B). To next analyze the effects of TNF‐α derived from GC tumors on the activation and B7‐H2 expression of/on neutrophils, we added TNF‐α blocking antibody into TTCS/neutrophil co‐culture, and TNF‐α blocking significantly inhibited neutrophil CD54 and B7‐H2 expression (Figure 4D; Figure S5C). Consistently, provision of exogenous TNF‐α into NTCS/neutrophil co‐culture increased neutrophil CD54 and B7‐H2 expression (Figure 4D; Figure S5C). Altogether, the data above suggest that TNF‐α derived from GC tumors induce the activation and B7‐H2 expression of/on neutrophils.

To analyze which pathways operate in the process of activating neutrophils and inducing neutrophil B7‐H2 expression, we pre‐processed blood neutrophils with relevant inhibitors and then cultured them with TTCS. We detected that only in the interdiction of ERK signal transduction or NF‐κB signal transduction the expressions of CD54 and B7‐H2 on TTCS‐conditioned neutrophils or TNF‐α‐stimulated neutrophils were inhibited (Figure 4E; Figure S5D and E). Moreover, p65, an ERK‐NF‐κB signaling pathway downstream substrate, was significantly more phosphorylated in TTCS‐conditioned neutrophils (Figure 4F) as well as in nucleus of TTCS‐conditioned neutrophils (Figure S6C), and these phosphorylations were abolished when we added TNF‐α blocking antibody, suggesting that ERK‐NF‐κB signaling pathway is important for the activation of neutrophils and neutrophil B7‐H2 expression by TNF‐α in tumors. Altogether, the findings above suggest that, in GC, TNF‐α can activate ERK‐NF‐κB signaling pathway to induce neutrophil activation and B7‐H2 expression.

### 3.5 Tumoral and tumor‐conditioned neutrophils induce protumorigenic IL‐17A‐producing Th subset polarization through a B7‐H2‐dependent manner

**FIGURE 5:**
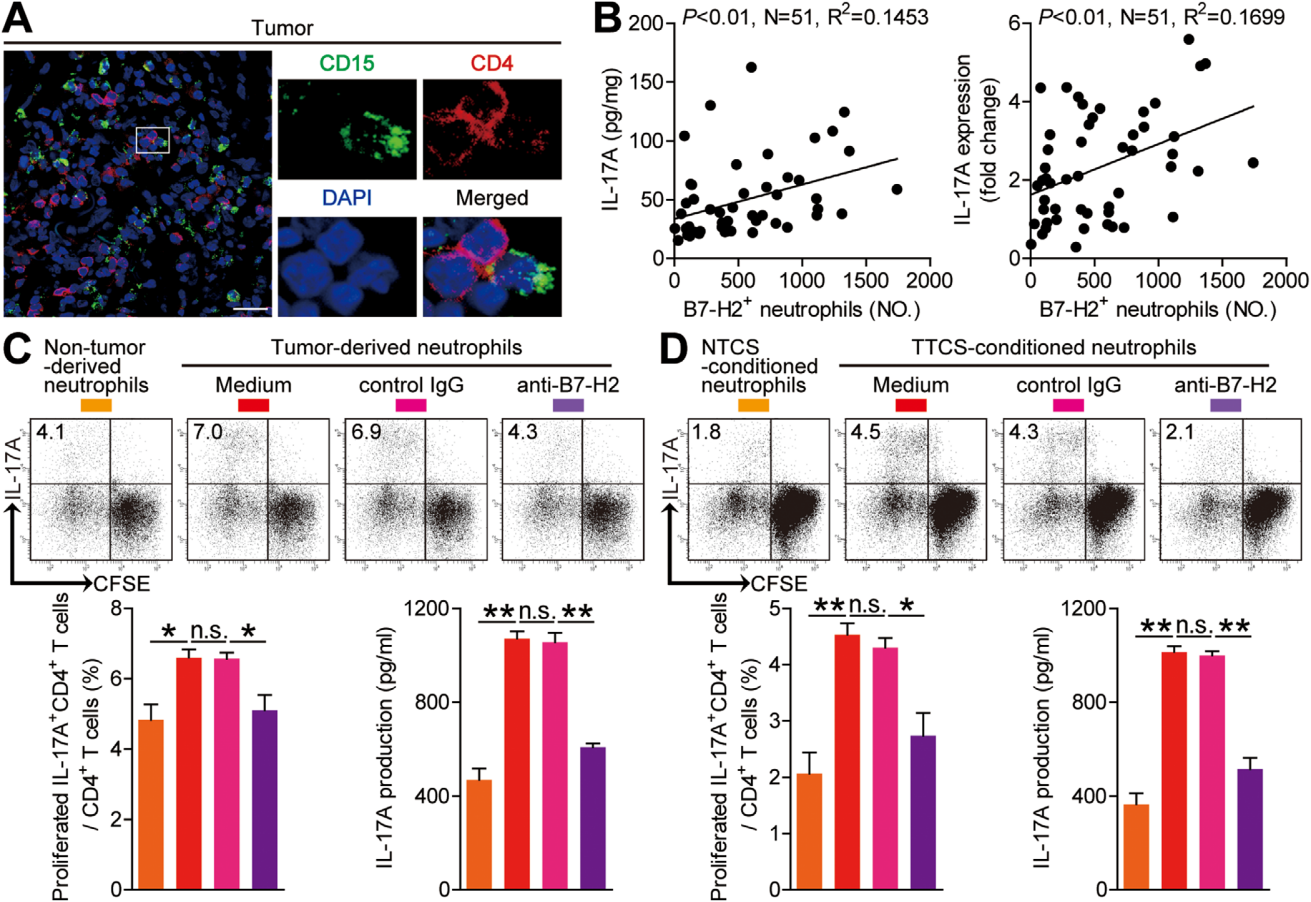
Tumor‐infiltrating and tumor‐conditioned neutrophils induce protumorigenic IL‐17A‐producing Th subset polarization through a B7‐H2‐dependent manner. (A) Representative analysis of CD15^+^ neutrophil (green) and CD4^+^ T cell (red) interactions in tumor tissues of GC patients by immunofluorescence. Scale bars: 20 microns. (B) The correlations between B7‐H2^+^ neutrophils and IL‐17A in human GC tumors were analyzed. Results are expressed as the number of B7‐H2^+^ neutrophils per million total cells and IL‐17A concentration or IL‐17A expression in tumor tissues. (C) CFSE‐labeled peripheral CD4^+^ T cells of GC patients were co‐cultured for 4 days with autologous neutrophils from non‐tumor or tumor tissues with or without anti‐B7‐H2 antibody. Representative data and statistical analysis of proliferated IL‐17A‐producing CD4^+^ T cells and IL‐17A production were shown (n = 3). (D) CFSE‐labeled peripheral CD4^+^ T cells of donors were co‐cultured for 4 days with autologous NTCS‐conditioned neutrophils or TTCS‐conditioned neutrophils with or without anti‐B7‐H2 antibody. Representative data and statistical analysis of proliferated IL‐17A‐producing CD4^+^ T cells and IL‐17A production were shown (n = 3). Each dot in panel (B) represents one patient. *P < 0.05; **P < 0.01; ^n.s.^ *P *> 0.05 for groups connected by horizontal lines

The interactions of CD15^+^ neutrophils with CD4^+^ T cells in GC tumor tissues (Figure 5A; Figure S7A), the positive correlations between neutrophils and CD4^+^ T cells in GC tumor tissues (Figure S7B), and the significant positive correlations between the levels of IL‐17A and B7‐H2^+^ neutrophils in GC tumor tissues (Figure 5B) imply that tumoral neutrophils may promote IL‐17A‐producing Th subset polarization via B7‐H2. Neutrophils from autologous tumor tissues and non‐tumor tissues were purified and co‐cultured with autologous blood CD4^+^ T cells. Tumoral neutrophils surpassed non‐tumor tissue neutrophils in inducting IL‐17A‐producing Th subset polarization (Figure 5C). Next, we added B7‐H2 blocking antibody into our tumoral neutrophil/blood CD4^+^ T‐cell co‐culture system, and observed that B7‐H2 blocking efficaciously attenuated such IL‐17A‐producing Th subset polarization mediated by tumoral neutrophils (Figure 5C). Altogether, the data above suggest that B7‐H2 contributes to tumoral neutrophil‐mediated IL‐17A‐producing Th subset polarization *ex vivo*.

Given the tumoral neutrophils induced IL‐17A‐producing Th subset polarization surpasses non‐tumor tissue neutrophils, we thus presumed that tumors might have effects on this process. Then we bead‐sorted peripheral CD4^+^ T cells and co‐cultured them with TTCS‐conditioned neutrophils or NTCS‐conditioned neutrophils, and found that TTCS‐conditioned neutrophils showed significantly more IL‐17A‐producing Th subset polarization (Figure 5D). To see whether B7‐H2 operates in this IL‐17A‐producing Th subset polarization, we added B7‐H2 blocking antibody in the CD4^+^ T cell/TTCS‐conditioned neutrophils co‐cultures. And we further found that blockade of B7‐H2 significantly attenuated such IL‐17A‐producing Th subset polarization mediated by TTCS‐conditioned neutrophils (Figure 5D). However, blocking B7‐H2 or blocking CD54 had no effects on the productions of IFN‐γ, IL‐4, or TGF‐β in our TTCS‐conditioned neutrophil‐CD4^+^ T cell co‐culture systems (Figure S7C), suggesting a specific induction of IL‐17A‐producing Th subset polarization and IL‐17A production. The data imply that neutrophils acquire the ability to induce IL‐17A‐producing Th subset polarization through B7‐H2 in GC tumors.

### 3.6 Tumor‐infiltrating and tumor‐conditioned neutrophils induce protumorigenic IL‐17A‐producing Th subset polarization through a B7‐H2‐ERK pathway

**FIGURE 6:**
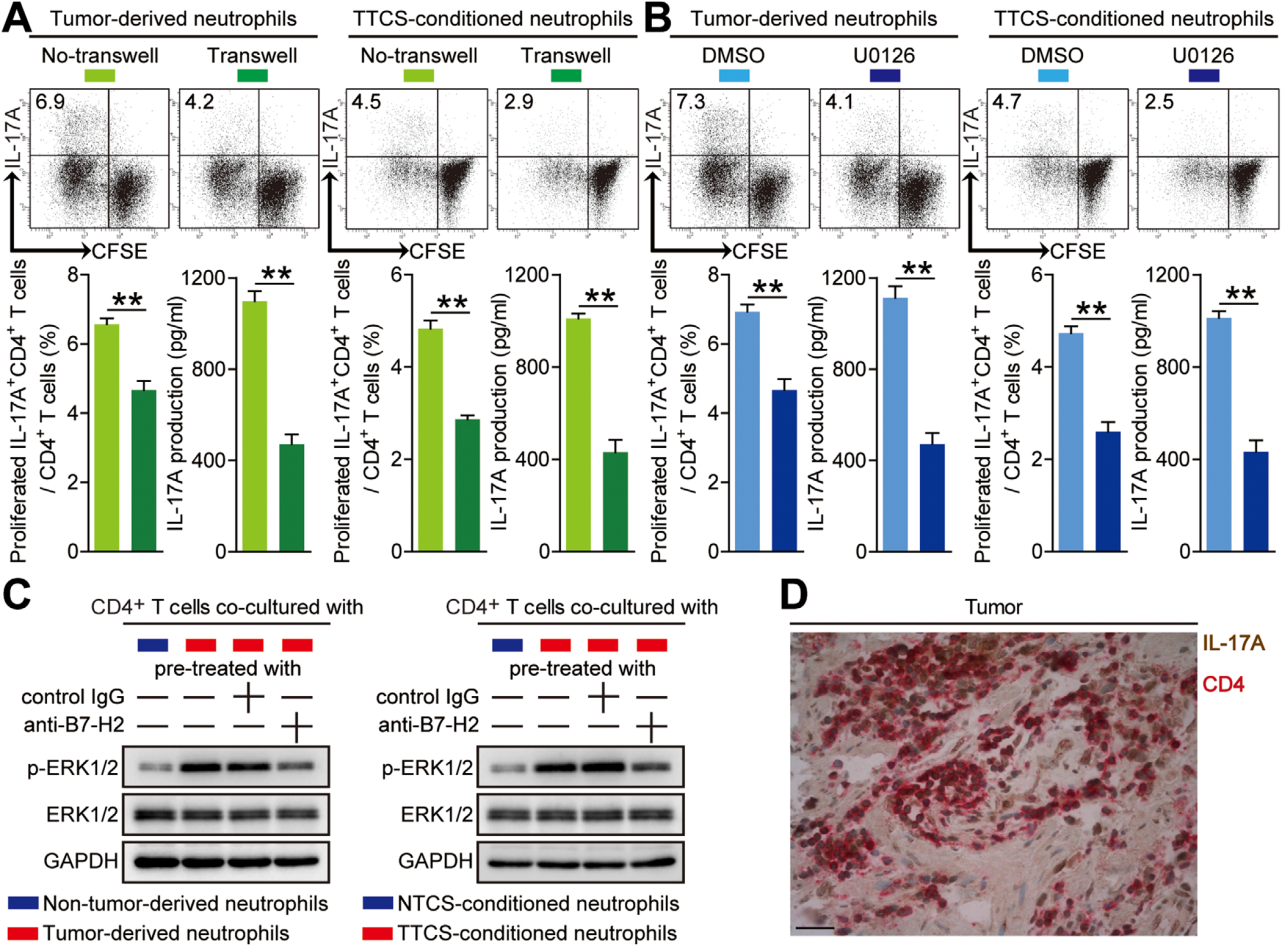
Tumor‐infiltrating and tumor‐conditioned neutrophils induce protumorigenic IL‐17A‐producing Th subset polarization through a B7‐H2‐ERK pathway. (A) CFSE‐labeled peripheral CD4^+^ T cells of GC patients or donors were co‐cultured for 4 days with autologous neutrophils from tumor tissues or autologous TTCS‐conditioned neutrophils with or without transwells. Representative data and statistical analysis of proliferated IL‐17A‐producing CD4^+^ T cells and IL‐17A production were shown (n = 3). (B) CFSE‐labeled peripheral CD4^+^ T cells of GC patients or donors pretreated with DMSO or U0126 were co‐cultured for 4 days with autologous neutrophils from tumor tissues or autologous TTCS‐conditioned neutrophils. Representative data and statistical analysis of proliferated IL‐17A‐producing CD4^+^ T cells and IL‐17A production were shown (n = 3). (C) Autologous neutrophils isolated from tumor or non‐tumor tissues, and TTCS‐ or NTCS‐conditioned neutrophils were pretreated with or without human B7‐H2 neutralizing antibody or control IgG (20 µg/ml) for 2 h. Then peripheral CD4^+^ T cells were co‐cultured with these neutrophils for 4 days. The ERK1/2 and p‐ERK1/2 proteins in CD4^+^ T cells were analyzed by western blot. (D) Representative analysis of IL‐17A‐expressing (brown) CD4^+^ cells (red) in tumor tissues of GC patients by immunohistochemical staining. Scale bars: 20 microns. *P < 0.05; **P < 0.01; ^n.s.^ *P *> 0.05 for groups connected by horizontal lines

To analyze the underlying mechanism of this induction more closely, transwell assays were performed, showing that cell‐cell contacts were needed for promoting IL‐17A‐producing Th subset polarization by tumor‐infiltrating neutrophils and TTCS‐conditioned neutrophils (Figure 6A), suggesting a surface B7‐H2, not a soluble B7‐H2 is acting in this induction of IL‐17A‐producing Th subset polarization.

It has also been shown that ERK pathway activation was involved in IL‐17A‐producing Th subset polarization,^21^, ^22^ to verify whether similar mechanisms might be involved in the induction of IL‐17A‐producing Th subset by neutrophils in GC, purified peripheral CD4^+^ T cells were first pre‐treated with U0126, the inhibitor of ERK pathway, and co‐cultured them with tumor‐infiltrating neutrophils and TTCS‐conditioned neutrophils for 4 days. As expected, blocking the signal transduction of ERK in CD4^+^ T cells efficiently attenuated such IL‐17A‐producing Th subset polarization mediated by tumor‐infiltrating neutrophils and TTCS‐conditioned neutrophils (Figure 6B). Moreover, ERK1/2, a downstream substrate of ERK signaling pathway, was more phosphorylated in CD4^+^ T cells co‐cultured with tumor‐infiltrating neutrophils and TTCS‐conditioned neutrophils than those co‐cultured with non‐tumor‐derived neutrophils and NTCS‐conditioned neutrophils, and this ERK1/2 phosphorylation was abolished when blocking B7‐H2 on tumor‐infiltrating neutrophils and TTCS‐conditioned neutrophils (Figure 6C). These results above indicate that ERK signaling pathway activation in CD4^+^ T cells induced by neutrophils'B7‐H2 is crucial for IL‐17A‐producing Th subset polarization in GC. Furthermore, IL‐17A expression was found on the majority of CD4^+^ cells in tumors analyzed (Figure 6D), suggesting that CD4^+^ cells were a dominant source of IL‐17A in the tumor tissues, and implying that IL‐17A‐producing Th subsets might have a potential role on GC.

### 3.7 Blockade of IL‐17A from tumor‐associated neutrophil‐polarized IL‐17A‐producing Th subsets inhibits the progression of GC tumors

**FIGURE 7:**
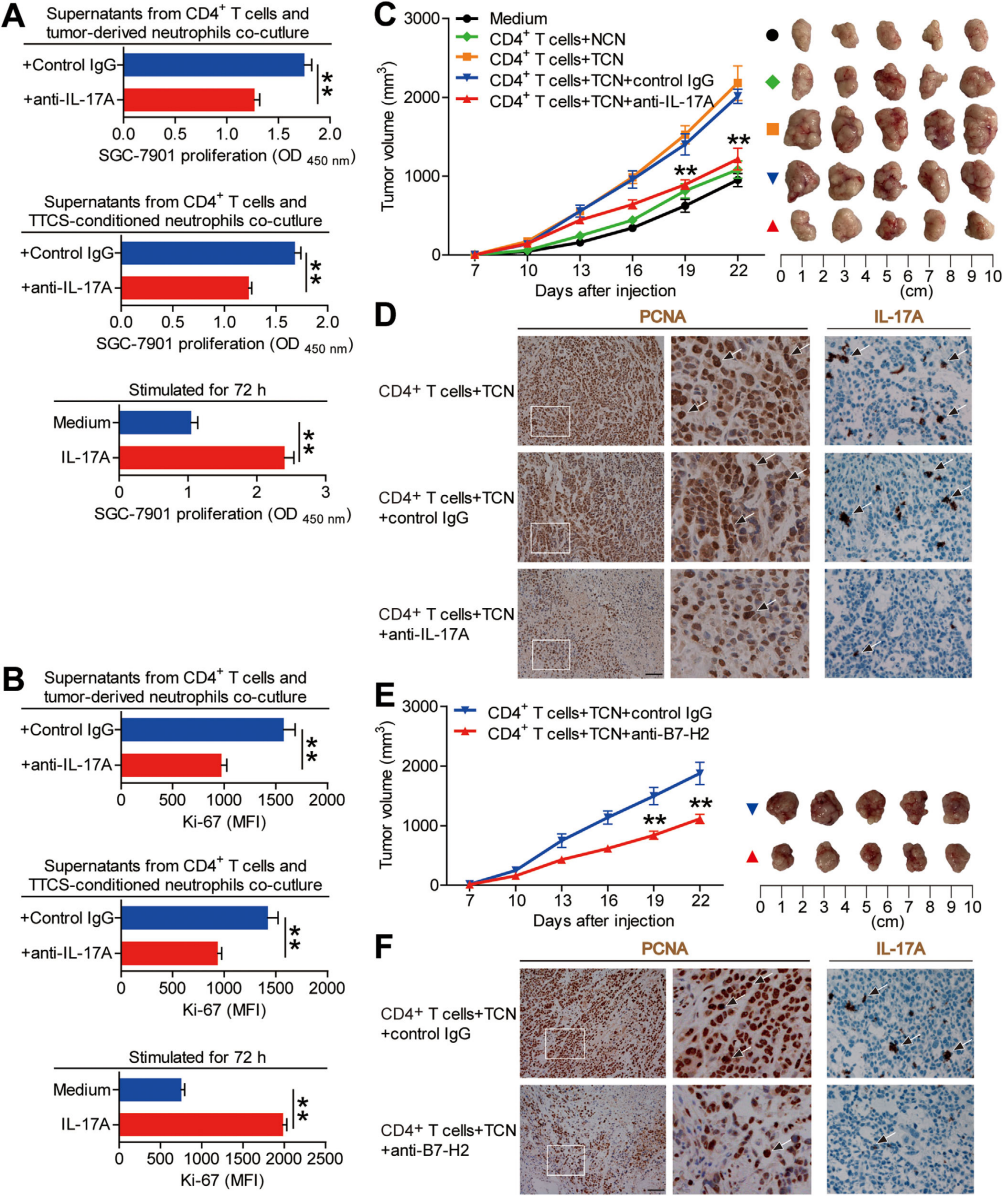
Blockade of IL‐17A from tumor‐associated neutrophil‐polarized IL‐17A‐producing Th subsets inhibits tumor growth and GC progression *in vivo*. (A and B) GC cells were stimulated with the culture supernatants from autologous peripheral CD4^+^ T cells and tumor‐derived neutrophils plus control IgG or IL‐17A neutralizing antibody, or the culture supernatants from autologous peripheral CD4^+^ T cells and TTCS‐conditioned neutrophils plus control IgG or IL‐17A neutralizing antibody, or exposed to IL‐17A as described in Methods. The proliferation of GC cells was analyzed by using CCK‐8 Kits (A) and Ki‐67 staining (B) (n = 3). **P < 0.01 for groups connected by horizontal lines. (C) Mice were injected with human SGC‐7901 cells, as described in Section 2. The control animals received no further injections. The experimental treatments entailed injections with CD4^+^ T cells in combination with NTCS‐conditioned neutrophils (NCN) or with CD4^+^ T cells in combination with TTCS‐conditioned neutrophils (TCN), followed sequentially injecting with IL‐17A blocking antibody or control IgG. The illustrated data represent tumor volumes (5 mice in each group). The day of tumor cell injection was counted as day 0. **P < 0.01, for groups injected with CD4^+^ T cells in combination with TCN and anti‐IL‐17A antibody, compared with groups injected with CD4^+^ T cells in combination with TCN and control IgG. The tumors were excised and photographed 22 days after injecting the tumor cells. (D) The proliferating cell nuclear antigen (PCNA) and IL‐17A expressions (brown) in tumors were analyzed. Scale bars: 100 microns. The arrowheads indicated PCNA positive or IL‐17A positive cells. (E) Mice were injected with human SGC‐7901 cells, as described in Methods. The experimental treatments entailed injections with CD4^+^ T cells in combination with TTCS‐conditioned neutrophils (TCN) plus B7‐H2 blocking antibody or control IgG. The illustrated data represent tumor volumes (5 mice in each group). The day of tumor cell injection was counted as day 0. **P < 0.01, for groups injected with CD4^+^ T cells in combination with TCN plus B7‐H2 blocking antibody, compared with groups injected with CD4^+^ T cells in combination with TCN plus control IgG. The tumors were excised and photographed 22 days after injecting the tumor cells. (F) The proliferating cell nuclear antigen (PCNA) and IL‐17A expressions (brown) in tumors were analyzed. Scale bars: 100 microns. The arrowheads indicated PCNA positive or IL‐17A positive cells

As for no IL‐17A expression in neutrophils in co‐culture systems above (Figure 7D), next, to analyze the effects of IL‐17A‐producing Th subset‐derived IL‐17A on GC cells *in vitro*, thus, we added IL‐17A blocking antibody in the culture supernatants from tumoral neutrophil/blood CD4^+^ T‐cell co‐culture system or the culture supernatants from CD4^+^ T cell/TTCS‐conditioned neutrophils co‐culture system which stimulated GC cells. Interestingly, by using CCK‐8 Kits (Figure 7A) and Ki‐67 staining (Figure 7B; Figure S7E), we found that blockade of IL‐17A significantly inhibited the proliferation of GC cells induced by those IL‐17A‐producing Th subset polarizing culture supernatants. In accordance with the above results, provision of exogenous IL‐17A effectively increased GC cell proliferation (Figure 7A and B; Figure S7E). Altogether, the data above suggest that IL‐17A‐producing Th subset‐derived IL‐17A promotes the proliferation of GC cells.

In order to analyze the tumor‐promoting effect of tumor‐associated neutrophil‐polarized IL‐17A‐producing Th subsets *in vivo*, we co‐cultured NTCS‐conditioned neutrophils (NCN) with CD4^+^ T cells, or TTCS‐conditioned neutrophils (TCN) with CD4^+^ T cells for 4 days and then injected them into the human NOD/SCID model mice bearing SGC‐7901‐derived tumor, then sequentially injected IL‐17A blocking antibody or control IgG every 2 days until the mice were sacrificed. As expected, compared to mice treated with CD4^+^ T cells plus NCN, mice transfused with CD4^+^ T cells plus TCN showed increased the growth of GC tumors as well as the progression of diseases (Figure 7C). Consistent with the key roles in promoting GC tumors of IL‐17A *in vivo*, mice transfused with CD4^+^ T cells plus TCN and IL‐17A neutralizing antibody showed reduced tumor volumes as well as GC progression at every time point from day 19, when compared to mice transfused with CD4^+^ T cells plus TCN and control IgG (Figure 7C). Furthermore, mice transfused with CD4^+^ T cells plus TCN and IL‐17A neutralizing antibody, showed decreased the proliferation of GC tumor cells as well as IL‐17A production (Figure 7D), compared to mice transfused with CD4^+^ T cells plus TCN and control IgG. As for no IL‐17A expression in neutrophils in co‐culture systems above (Figure S7D), the data above indicate that IL‐17A from tumor‐associated neutrophil‐polarized IL‐17A‐producing Th subsets contributes to the progression of GC tumors *in vivo*. Furthermore, mice treated with CD4^+^ T cells plus TCN and B7‐H2 neutralizing antibody, showed decreased the growth of GC tumors as well as the progression of diseases (Figure 7E) as well as decreased the proliferation of GC tumor cells and IL‐17A production (Figure 7F), compared to mice transfused with CD4^+^ T cells plus TCN and control IgG.

### 3.8 B7‐H2^+^ neutrophils and IL‐17A are closely associated with GC tumor progression and poor patient prognosis

**FIGURE 8:**
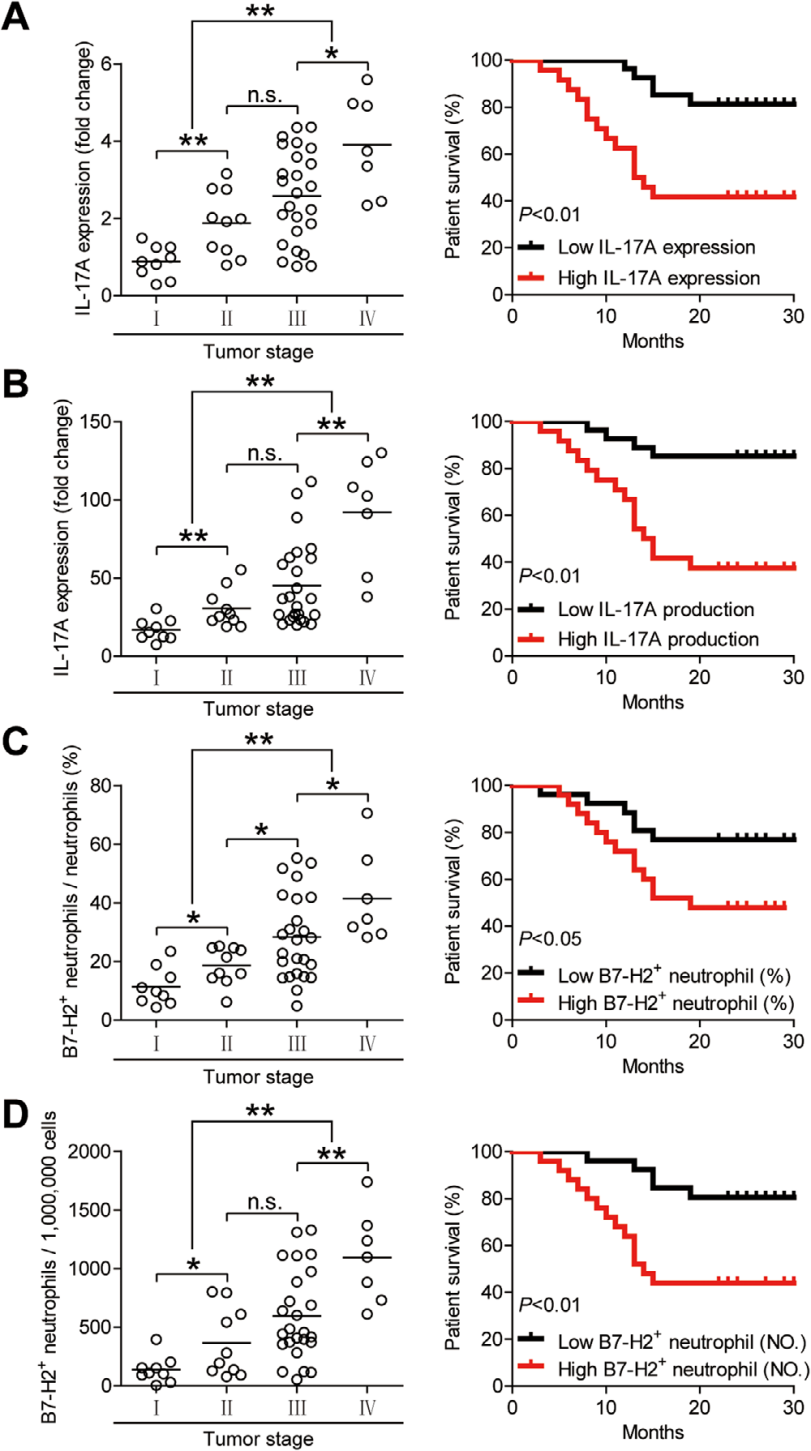
B7‐H2^+^ neutrophils correlate with advanced tumor stage and poor survival in patients with GC. (A) Intratumoral IL‐17A expression among TNM stages was compared. Kaplan‐Meier plots for overall survival by median IL‐17A expression (2.1‐fold change). (B) Intratumoral IL‐17A production among TNM stages was compared. Kaplan‐Meier plots for overall survival by median IL‐17A production (30.01721 pg/mg). (C) Intratumoral B7‐H2^+^ neutrophil percentage in total neutrophils (%) among TNM stages was compared. Kaplan‐Meier plots for overall survival by median B7‐H2^+^ neutrophil percentage (22.8). Results are analyzed as the percentage of B7‐H2^+^ neutrophils in total neutrophils in tumor tissues. (D) Intratumoral B7‐H2^+^ neutrophil number per million total cells (NO.) among TNM stages was compared. Kaplan‐Meier plots for overall survival by median B7‐H2^+^ neutrophil number (416). Results are analyzed as the number of B7‐H2^+^ neutrophils per million total cells in tumor tissues. The horizontal bars in panels (A–D) represent mean values. Each ring in panels (A–D) represents one patient. *P < 0.05; **P < 0.01; ^n.s.^ *P *> 0.05 for groups connected by horizontal lines

Finally, we analyzed the clinical associations as well as the prognosis of inflammatory molecule IL‐17A and B7‐H2^+^ neutrophils in advanced GC patients. First, the expression and production of IL‐17A in advanced GC patients were significantly increased than that in early GC patients (Figure 8A and B). In the next place, compared GC patients with high versus low expression and production of IL‐17A, the survival rates of 30 months were significantly lower for those GC patients with the higher level of IL‐17A expression and production (Figure 8A and B). Furthermore, the lower 30‐month overall survival rates for higher IL‐17A expression (Figure S8A) and production (Figure S8B) were more pronounced in the GC patients with TNM stage (III+IV). Similar findings were obtained when the patient cohort was stratified based on the percentage (Figure 8C; Figure S8C) and the number (Figure 8D; Figure S8D) of intratumoral B7‐H2^+^ neutrophils. Moreover, we also found that increased IL‐17A expression (Figure S9A) and IL‐17A production (Figure S9B) were closely correlated with advanced tumor stage and increased tumor size. Similar findings were obtained when the patient cohort was stratified based on the percentage (Figure S10A) and the number (Figure S10B) of intratumoral B7‐H2^+^ neutrophils. Importantly, the Cox proportional hazard model‐based multivariate analyses showed that intratumoral B7‐H2^+^ neutrophils and IL‐17A could predict poor patient prognosis (Table S4). Altogether, the findings suggest that IL‐17A and B7‐H2^+^ neutrophils are closely associated with GC tumor progression and poor patient prognosis.

## 4 DISCUSSION

Although it is generally accepted the notion of strong immune suppression in tumors of cancer patients, substantial evidences show that inflammatory reactions in cancers can promote disease progression.^23^, ^24^ Our previous data revealed that proinflammatory IL‐17A‐producing Th cells were enriched predominantly in the blood of GC patients,^15^ here, our present study have further demonstrated that, in GC tumor tissues, activated neutrophils induce IL‐17A‐producing Th subset polarization through TNF‐α‐B7‐H2‐dependent pathway, and that subsequently IL‐17A‐producing Th subsets exert protumorigenic effects through inflammatory molecule IL‐17A. From the current research, we have discovered and confirmed for the first time that there are neutrophils with high expression of B7‐H2 in GC tissues and are significantly related to the prognosis of GC patients; it is also the first authentication for tumor‐derived TNF‐α to activate neutrophils which polarize protumorigenic IL‐17A‐producing Th subsets linking mechanistically the pathological roles of B7‐H2^+^ neutrophils in GC environment.

Elucidating the potential interactions between innate and adaptive immune cells in the tumor environments is important for investigating the progression of cancers.^25^ As an abundant population of adaptive immune cells in tumors,^11^ although many previous reports have investigated multiple functions of T cells in GC tumors,^26^ the functional polarization of IL‐17A‐producing Th subsets especially connecting with neutrophils, the most abundant innate immune cells, remains unknown. Now our findings support a new notion that neutrophils exposed to tumor‐derived TNF‐α that activates signals associated with B7‐H2 expression, have displayed a potential to generate inflammatory IL‐17A‐producing Th subsets in GC milieu. Moreover, according to our results, we think that neutrophils and B7‐H2 are expected to be one of the main drivers of IL‐17A production by CD4^+^ T cells.

Neutrophils have been investigated in human GC for many years. However, there is presently unknown about these tumor‐infiltrating neutrophils’ accumulation and phenotype. Recently, neutrophils were shown to may be attracted into GC environment in a CXC chemokine‐dependent manner,^27^ although the detailed mechanisms were not clarified. In this study, we have now identified a definitive mechanism for neutrophil chemotaxis; tumor tissues can produce more CXCL6/CXCL8 when compared to non‐tumor tissues, which attracts neutrophil migration via CXCL6/CXCL8‐CXCR1 interactions, suggesting that CXCL6/CXCL8‐CXCR1 axis plays important roles in the migration of neutrophils in GC. Although it has been shown that, in GC patients, neutrophils in peripheral blood^9^ and tumor tissues^10^ are significantly elevated, here, we have expanded the phenotype of tumor‐infiltrating neutrophils upon these previous observations. Although CD66b is also expressed by eosinophils, we have found that CD15 and CD66b are co‐expressed on the majority of neutrophils in GC tumors. Furthermore, our profiling of tumoral neutrophils reveals that they differ from their blood counterparts both phenotypically and functionally. Tumoral neutrophils exhibit a more activated phenotype characterized by the upregulation of activating molecule CD54.^9^ Most interestingly, these tumoral neutrophils also show positive expressions between CD54 and B7‐H2, indicating a potential role of B7‐H2 on activated neutrophils in GC. Recently, it has been reported that N1 and N2 neutrophils exist in solid tumors.^28^ N2 neutrophils promote tumor progression mostly via the secretion of inflammatory or angiogenic factors including iNOS, PEG2, or ROS,^29^ implying that B7‐H2 over‐expression is unlikely to be the only alteration in GC‐associated neutrophils.

Th cells are one of the most important cellular components in tumors,^30^ but investigation is limited for the pathological functions of Th cells in promoting tumor progression or with respect to the underlying mechanisms involved in regulating that process. In this study, we have identified a pathological function of neutrophils, particularly activated neutrophils, in generating proinflammatory IL‐17A‐producing Th subsets undergoing B7‐H2 triggering. ICOSL (also called B7‐H2)/ICOS (inducible T‐cell co‐stimulator) axis‐elicited sequential signal activation is essential for the generation of several Th subsets.^31^, ^32^ It has been widely proved that B7‐H2 expression on acute myeloid leukemia cells,^33^ bone marrow‐derived mesenchymal stem cells,^34^ plasmacytoid dendritic cells,^35^ and melanoma cells^36^ can promote the activation and expansion of regulatory T cells (Tregs), which contributes to immune evasion or immune suppression in tumor environments. However, in gastric mucosa, downregulation of B7‐H2 expression on gastric epithelial cells by *Helicobacter pylori* infection could inhibit Th17 responses,^37^ suggesting that B7‐H2 is involved in promoting IL‐17A‐producing Th subsets. Our data in this study are similar to the latter reports exhibiting a promoting role of neutrophils on the polarization of IL‐17A‐producing Th subsets dependent of B7‐H2 in GC. More important, within *in vivo* GC models, our hypothesis that tumor‐activated B7‐H2^+^ neutrophils induce IL‐17A‐producing Th subset response and promote the progression of GC tumors has been fully verified. Moreover, we have demonstrated that these protumorigenic effects are through inflammatory molecule IL‐17A from IL‐17A‐producing Th subsets as blocking IL‐17A can reverse it. It may be speculated that, in the different cancers, B7‐H2‐expressing profile on different cell types may have different inducing functions on Th subset polarization, which probably orchestrates immunosuppressive or inflammatory tumor milieu. Furthermore, as for the promoting effects of IL‐17A on GC in our studies, IL‐17A induction may have negative impacts on anti‐tumor immune responses, and the impacts of B7‐H2 ligation to ICOS on Tregs in tumor immunosurveillance need further investigation. Additionally, as for the contaminant cells in co‐culture systems, we think that they may be apoptotic neutrophils, which may have little effect on T‐cell polarization.

B7‐H2 is expressed on many cell types and is shown to be over‐expressed connecting with immune activation, in cases with inflammations or tumors.^38^ Previous studies have shown that B7‐H2 up‐regulation on mesenchymal stem cells can be induced by TNF‐α.^39^ Now, our team has also identified that tumoral TNF‐α induces neutrophil B7‐H2 expression in human GC. As for that TNF‐α has only a modest effect relative to conditioned media, it is likely that there are other factors in conditioned media from tumor cells that contribute to induction of CD54 and B7‐H2 on neutrophils. Besides, research has revealed that TNF‐α up‐regulates B7‐H2 on CD34^+^ progenitor cells by NF‐κB activation,^40^ which resembles our data in this study on the regulation B7‐H2 via the activation of ERK‐NF‐κB signaling pathways induced by TNF‐α in GC‐associated neutrophils. Here, we also have verified TNF‐α as a crucial proinflammatory factor in tumor tissues, which can be derived from tumors of GC patients to activate neutrophils besides inducing the expression of B7‐H2, suggesting relative importance of TNF‐α in neutrophil activation and tumorigenesis. Additionally, as for the differences in the effects of neutrophils or GC cells upon stimulation, we think that the reason is due to that recombinant proteins (including TNF‐α and IL‐17A) may have less biological activity than the naturally produced ligand.

**FIGURE 9:**
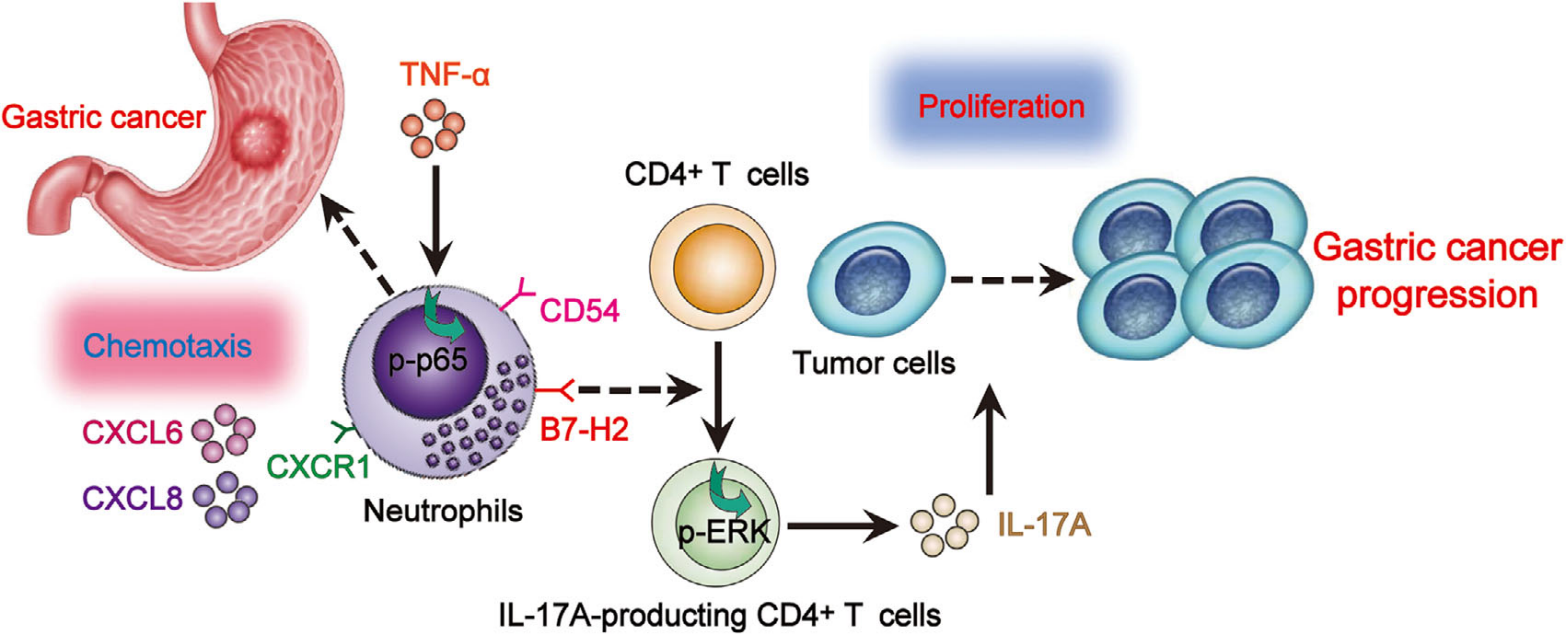
A proposed model of cross‐talks among neutrophils, CD4^+^ T cells, and tumor cells leading to neutrophil‐mediated protumorigenic IL‐17A‐producing Th subset polarization and tumor progression in GC environment. CXCL6/CXCL8‐CXCR1 chemotaxis mediates the recruitment and accumulation of neutrophils into GC environment, which upregulate CD54 and B7‐H2 expression via ERK‐NF‐κB signaling pathway activation by tumor‐derived TNF‐α. Neutrophils polarize IL‐17A‐producing Th subsets in a B7‐H2‐dependent manner; these polarized IL‐17A‐producing Th cells exert protumorigenic roles via inflammatory molecule IL‐17A, which contributed to the growth and progression of human GC

In a word, here we propose a model involving interplay between neutrophils, CD4^+^ T cells, and tumor cells in the GC tumor environments (Figure 9). First, CXCL6/CXCL8‐CXCR1 chemotaxis axis mediates the accumulation of neutrophils in GC tumors. Secondly, GC tumor‐derived TNF‐α promotes the activation and B7‐H2 expression of/on neutrophils via ERK‐NF‐κB signaling pathway activation. Finally, neutrophils polarize IL‐17A‐producing Th subsets through B7‐H2‐dependent pathway, which exerts protumorigenic effects mostly by promoting GC cell proliferation via IL‐17A, contributing to the progression of GC tumors. In conclusion, our results illuminate pathogenic roles of neutrophils in GC with a novel mechanism that TNF‐α‐activated neutrophils link B7‐H2 to protumorigenic IL‐17A‐producing Th subset polarization in human GC milieu. Thus, blocking these pathological TNF‐α‐B7‐H2‐IL‐17A pathways may be served as novel therapeutic strategies for GC treating.

## ETHICS APPROVAL AND CONSENT TO PARTICIPATE

The study was approved by the Ethics Committee of Qijiang Hospital of the First Affiliated Hospital of Chongqing Medical University and the Southwest Hospital of Third Military Medical University. Each subject provided written informed consent.

## CONFLICT OF INTEREST

The authors declare that there are no conflict of interest.

## AUTHOR CONTRIBUTIONS

Study concept and design and drafting of the manuscript: Yuan Zhuang. Acquisition of data and analysis and interpretation of data: Yuan Zhuang, Zhi‐guo Shan, Jun Chen, and Jin‐shan Liu. Critical revision of the manuscript for important intellectual content: Yuan Zhuang, Yong‐liang Zhao, Liu‐sheng Peng, and Jin‐shan Liu. Statistical analysis: Yuan Zhuang, Yong‐liang Zhao, Zhi‐guo Shan, Jun Chen, Jin‐yu Zhang, Ting‐ting Wang, Liu‐sheng Peng, Yong‐sheng Teng, Fang‐yuan Mao, and Ping Cheng. Obtained funding: Yuan Zhuang, Jin‐yu Zhang, Fang‐yuan Mao, Yong‐liang Zhao, and Wei‐ying Zhou. Technical or material support: Yong‐liang Zhao, Liu‐sheng Peng, Jin‐shan Liu, Jun Chen, and Quan‐ming Zou.

## Supporting information

Supporting information.

Supporting information.

## Data Availability

The data that support the findings of this study are available from the corresponding author upon reasonable request.
