# Supplementary material for: Activated neutrophils polarize protumorigenic interleukin‐17A‐producing T helper subsets through TNF‐α‐B7‐H2‐dependent pathway in human gastric cancer
Source: Clin Transl Med. 2021 Jun 27;11(6):e484. doi: 10.1002/ctm2.484 (PMC8236123; doi:10.1002/ctm2.484)
Supplement: Supplementary file 1 — Supporting information. [file CTM2-11-e484-s002.pdf]

Supplementary Figure 1.

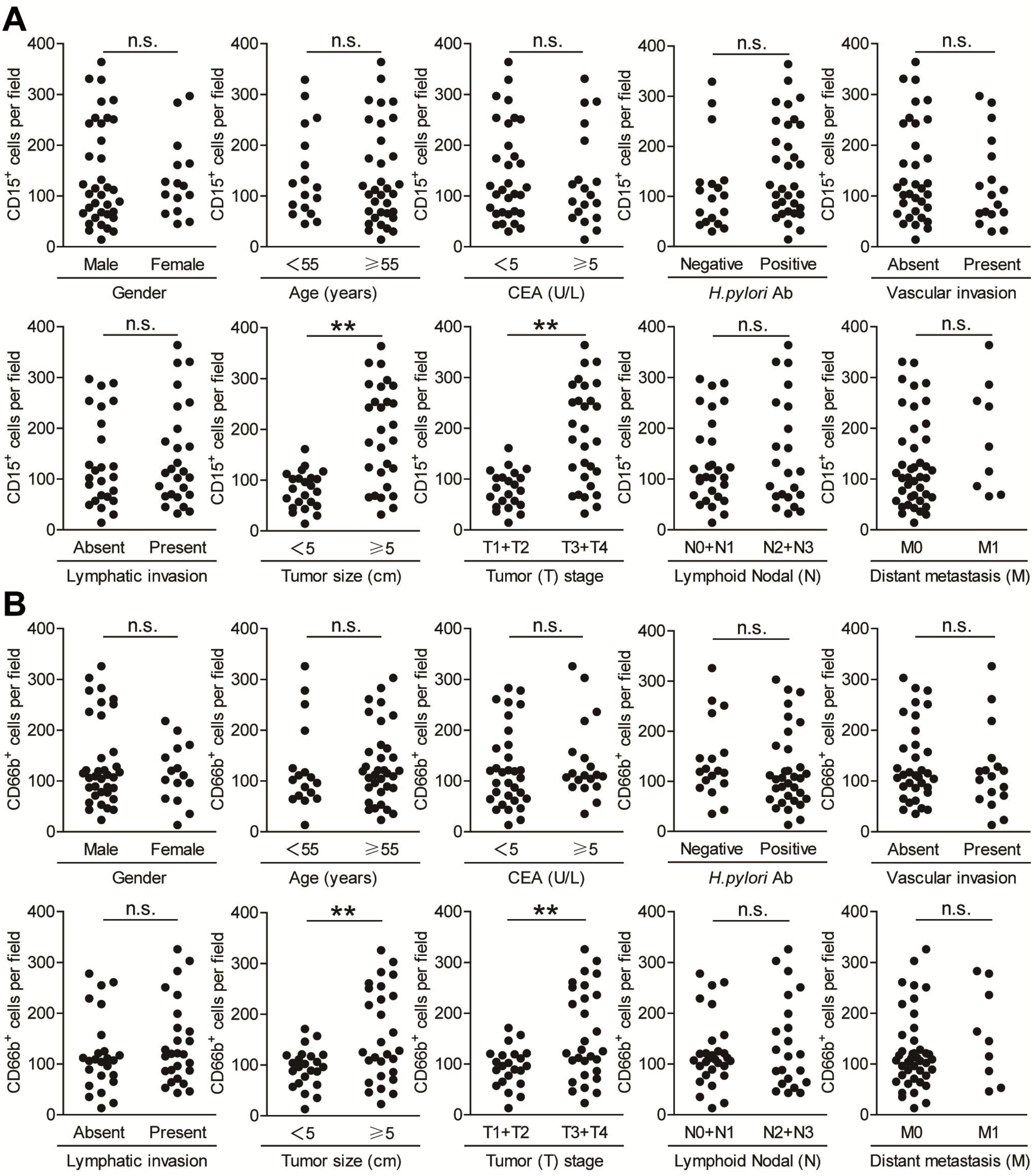

Supplementary Figure 1. $CD15^+$ neutrophil number (A) or $CD66b^+$ neutrophil number (B) and their potential correlations with clinical parameters. Neutrophil number was analyzed for correlations with clinical pathological parameters. **, P<0.01; n.s., P>0.05 for groups connected by horizontal lines. Each dot represents 1 patient. CEA, carcinoembryonic antigen; *H.pylori* Ab, *Helicobacter pylori* antibody.

Supplementary Figure 2.

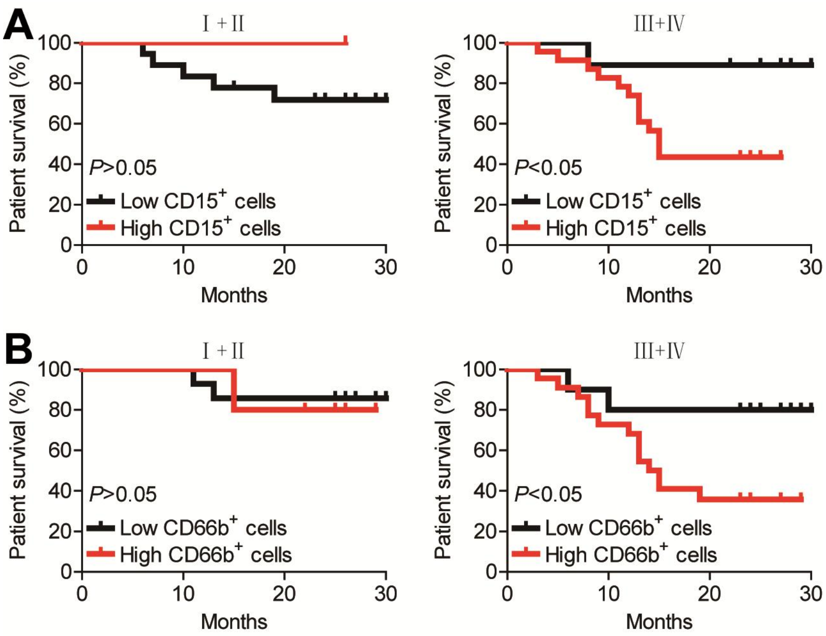

Supplementary Figure 2. Increased neutrophil accumulation in GC tumors predicts poor patient survival. (A) Kaplan-Meier plots for overall survival of the GC patients with TNM stage (I+II) or with TNM stage (III+IV) by median $CD15^+$ neutrophil number respectively. (B) Kaplan-Meier plots for overall survival of the GC patients with TNM stage (I+II) or with TNM stage (III+IV) by median $CD66b^+$ neutrophil number respectively.

Supplementary Figure 3.

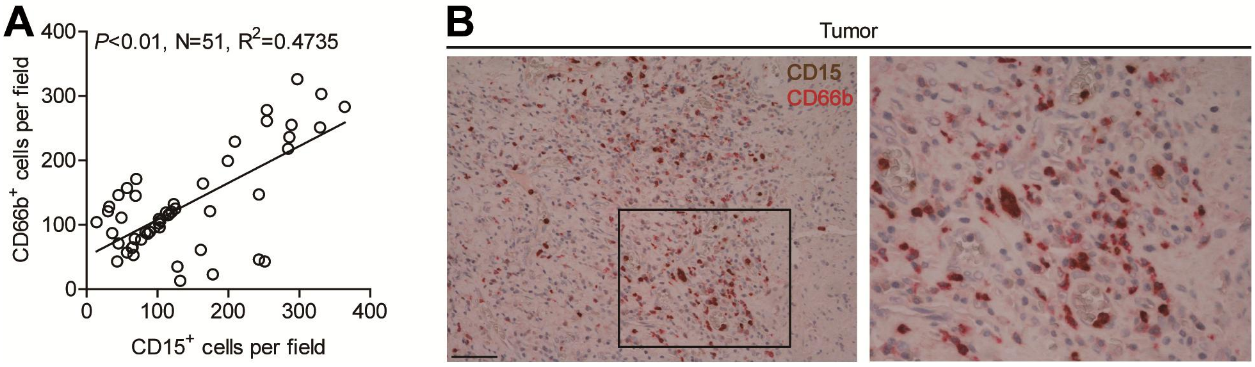

Supplementary Figure 3. CD15⁺ neutrophils and CD66b⁺ neutrophils are correlated in GC tumors. (A) The correlations between CD15⁺ cells and CD66b⁺ cells in human GC tumors were analyzed. Results are expressed as the number of CD15⁺ cells per field and CD66b⁺ cells per field in tumor tissues by immunohistochemical staining and counting. Each ring represents 1 patient. (B) Representative analysis of co-expression of CD15 (brown) and CD66b (red) on cells in tumor tissues of GC patients by immunohistochemical staining. Scale bars: 100 microns.

Supplementary Figure 4.

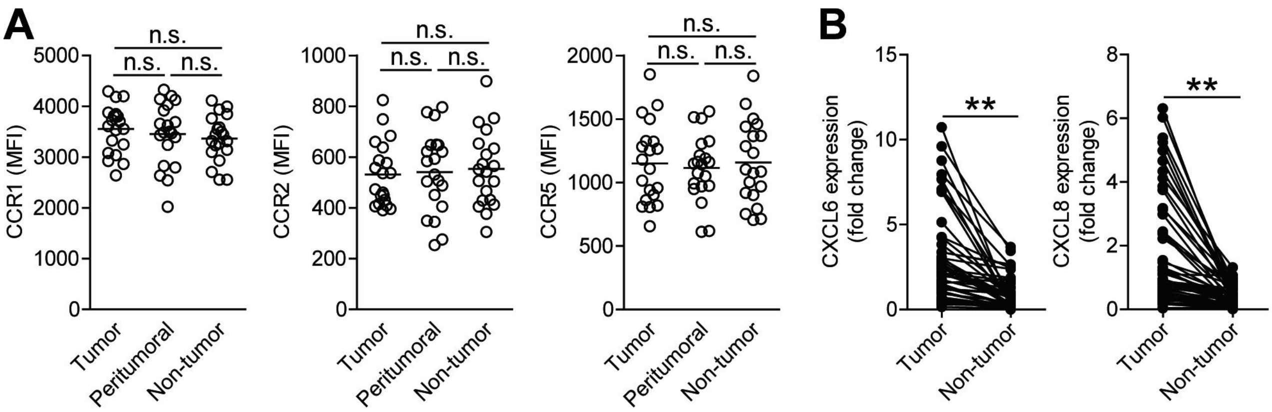

Supplementary Figure 4. Increased neutrophil accumulation is promoted by CXCL6/CXCL8-CXCR1-mediated chemotaxis. (A) Statistics analysis of the expression of CCR1, CCR2 and CCR5 on neutrophils in each samples of patients with GC (n=20). (B) CXCL6 and CXCL8 expression between autologous tumor and non-tumor tissues (n=51) was analyzed. *, $P<0.05$; **, $P<0.01$, n.s., $P>0.05$ for groups connected by horizontal lines.

Supplementary Figure 5.

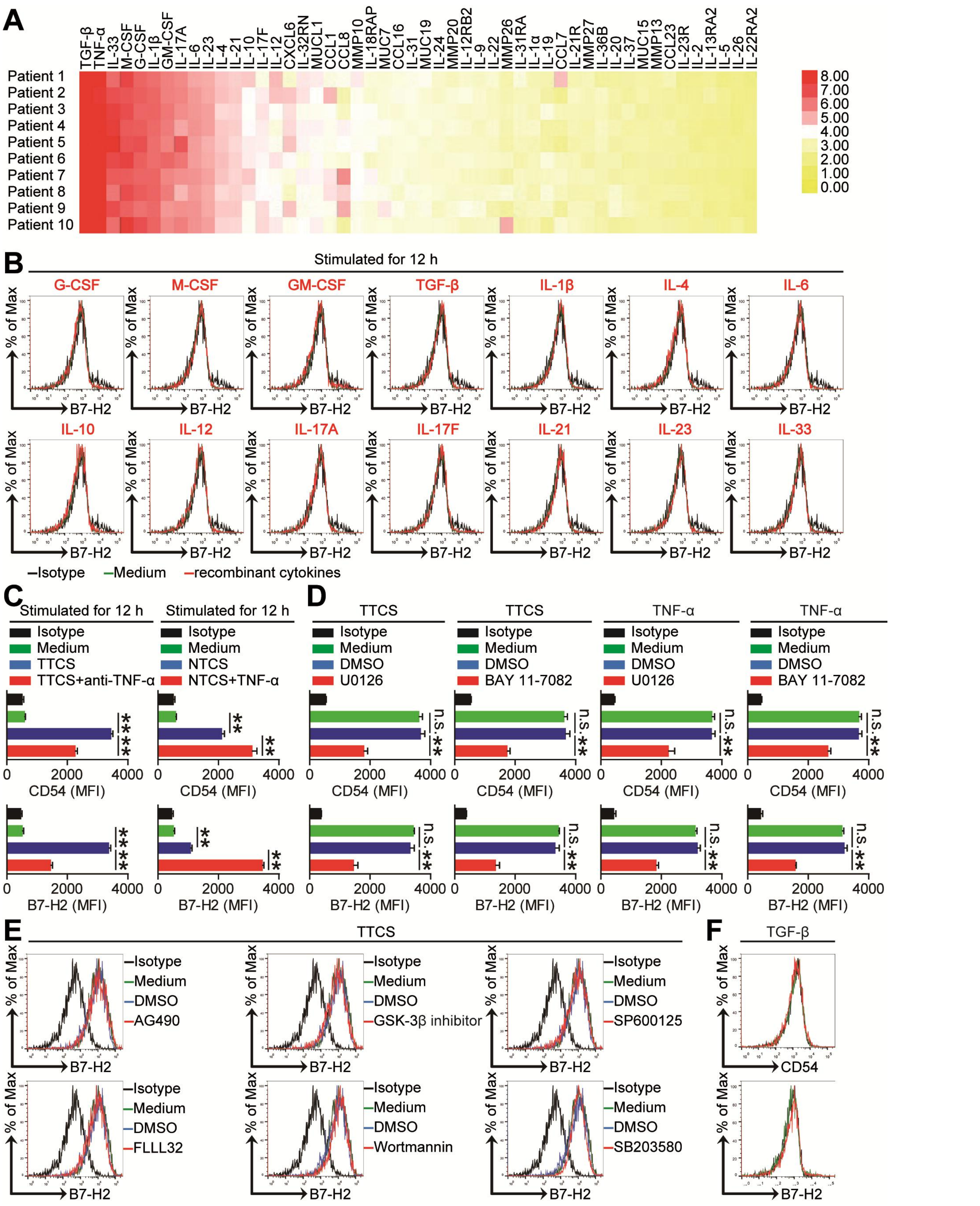

Supplementary Figure 5. Tumor-derived TNF-α activates neutrophils and induces B7-H2 expression on neutrophils via ERK-NF-κB pathway. (A) Clustering of microarray data for the expression of 50 pro-inflammatory cytokine genes in human tumor tissues from 10 GC patients. (B) Expression of B7-H2 on

neutrophils exposed to G-CSF, M-CSF, GM-CSF, TGF-β, IL-1β, IL-4, IL-6, IL-10, IL-12, IL-17A, IL-17F, IL-21, IL-23, IL-33 (100 ng/ml) for 12 hours. black, isotype control. (C) Statistical analysis of the expression of CD54 and B7-H2 on neutrophils exposed to TTCS with anti-TNF-α antibody or NTCS with TNF-α for 12 hours (n=3). black, isotype control. (D) Statistical analysis of the expression of CD54 and B7-H2 on neutrophils exposed to TTCS or TNF-α with or without U0126 (an ERK inhibitor) or BAY 11-7082 (an IκBα inhibitor) for 12 hours (n=3). black, isotype control. (E) Expression of B7-H2 on neutrophils exposed to 50% TTCS with or without AG490 (a JAK inhibitor), SP600125 (a JNK inhibitor), FLLL32 (an STAT3 inhibitor), Wortmannin (a PI3K inhibitor), SB203580 (an MAPK inhibitor), or GSK-3β inhibitor for 12 hours. black, isotype control. (F) Expression of B7-H2 and CD54 on neutrophils exposed to TGF-β (100 ng/ml) for 12 hours. black, isotype control.

Supplementary Figure 6.

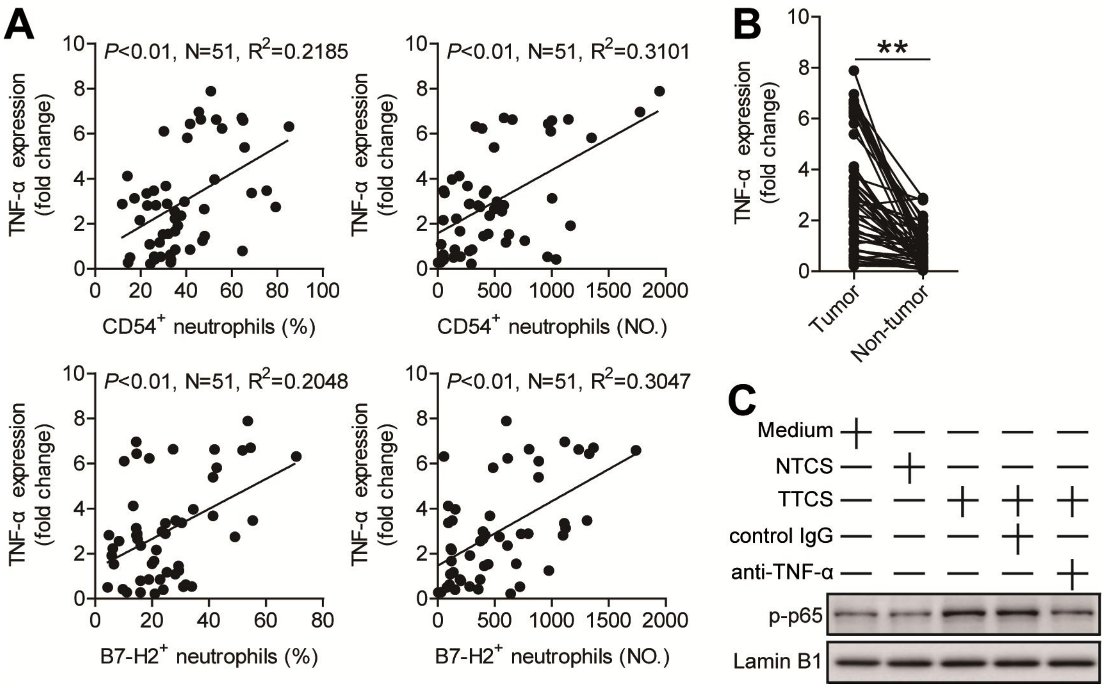

Supplementary Figure 6. Tumor-derived TNF-α activates neutrophils and induces B7-H2 expression. (A) The correlations between TNF-α and CD54$^+$ neutrophils or B7-H2$^+$ neutrophils in human tumors were analyzed. Results are expressed as percentage of CD54$^+$ neutrophils and B7-H2$^+$ neutrophils in total neutrophils or the number of CD54$^+$ neutrophils and B7-H2$^+$ neutrophils per million total cells and TNF-α expression in tumor tissues. (B) TNF-α expression between autologous tumor and non-tumor tissues (n=51) was analyzed. (C) The p-p65 proteins in nucleus of neutrophils exposed to autologous TTCS, NTCS, or TTCS with anti-TNF-α antibody or control IgG for 12 hours were analyzed by western blot. Each dot in panels A or B represents 1 patient. **, *P*<0.01 for groups connected by horizontal lines.

Supplementary Figure 7.

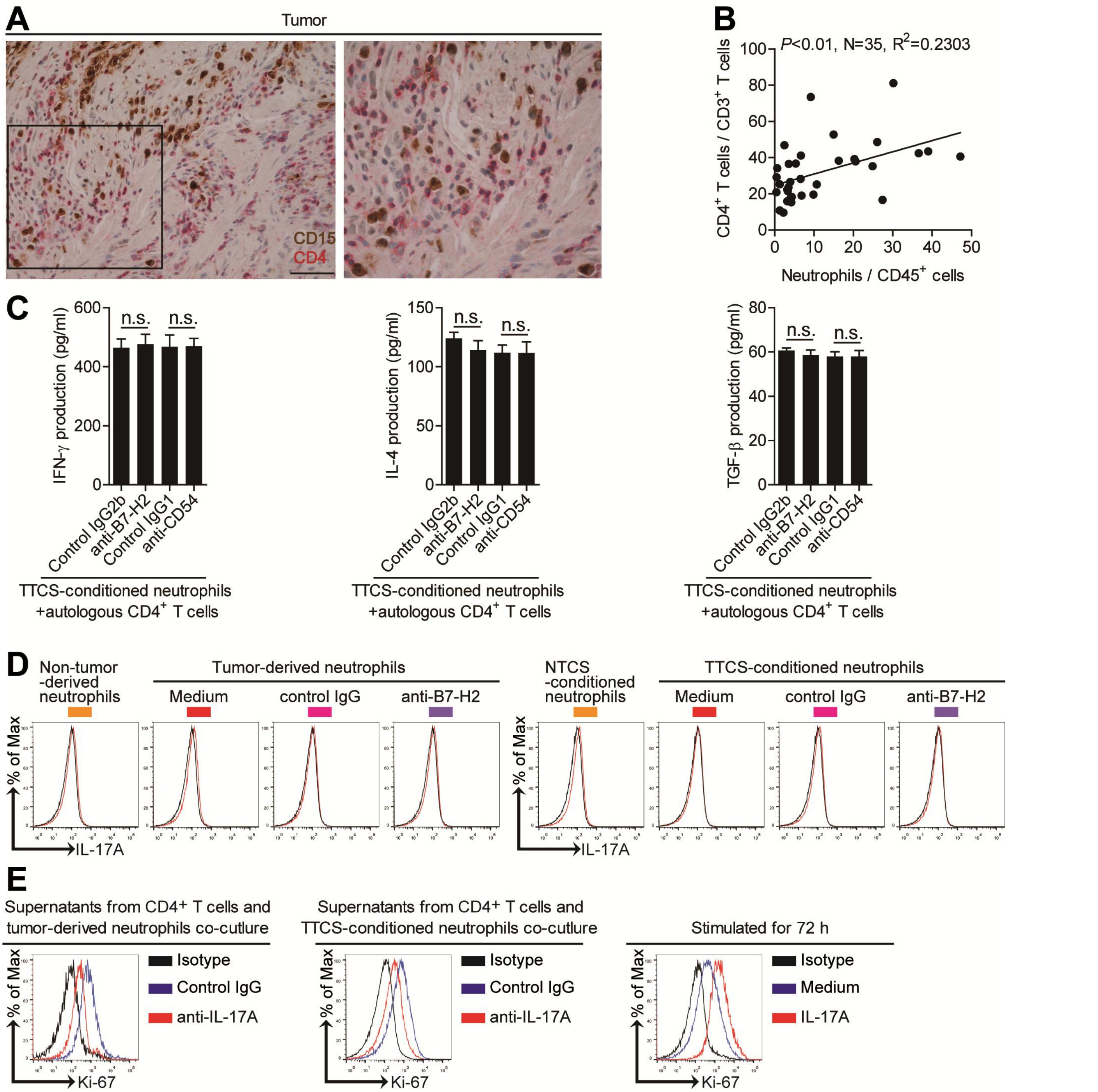

Supplementary Figure 7. Tumor-infiltrating and tumor-conditioned neutrophils induce protumorigenic IL-17A-producing Th subset polarization through a B7-H2-dependent manner, which promotes tumor growth and GC progression *in vivo*. (A) Representative analysis of CD15$^+$ neutrophil (brown) and CD4$^+$ T cell (red) interactions in tumor tissues of GC patients by immunohistochemical staining. Scale bars: 100 microns. (B) The correlations between neutrophils and CD4$^+$ T cells in human GC tumors were analyzed. Results are expressed as the percentage of neutrophils in CD45$^+$ cells and the percentage of CD4$^+$ T cells in CD3$^+$ T cells in tumor tissues. (C) CFSE-labeled peripheral CD4$^+$ T cells of donors were co-cultured for 4 days with autologous TTCS-conditioned neutrophils with or without anti-B7-H2 or anti-CD54 antibody. Statistical

analysis of the production of IFN-γ, IL-4 and TGF-β was shown (n=3). (D) CFSE-labeled peripheral $CD4^+$ T cells of GC patients or of donors were co-cultured for 4 days with autologous neutrophils from non-tumor or tumor tissues or with autologous NTCS-conditioned neutrophils or TTCS-conditioned neutrophils with or without anti-B7-H2 antibody. Representative data of IL-17A expression in these neutrophils was shown. (E) GC cells were stimulated with the culture supernatants from autologous peripheral $CD4^+$ T cells and tumor-derived neutrophils plus control IgG or IL-17A neutralizing antibody, or the culture supernatants from autologous peripheral $CD4^+$ T cells and TTCS-conditioned neutrophils plus control IgG or IL-17A neutralizing antibody, or exposed to IL-17A as described in Methods. The proliferation of GC cells was analyzed by using Ki-67 staining. n.s., $P>0.05$ for groups connected by horizontal lines.

Supplementary Figure 8.

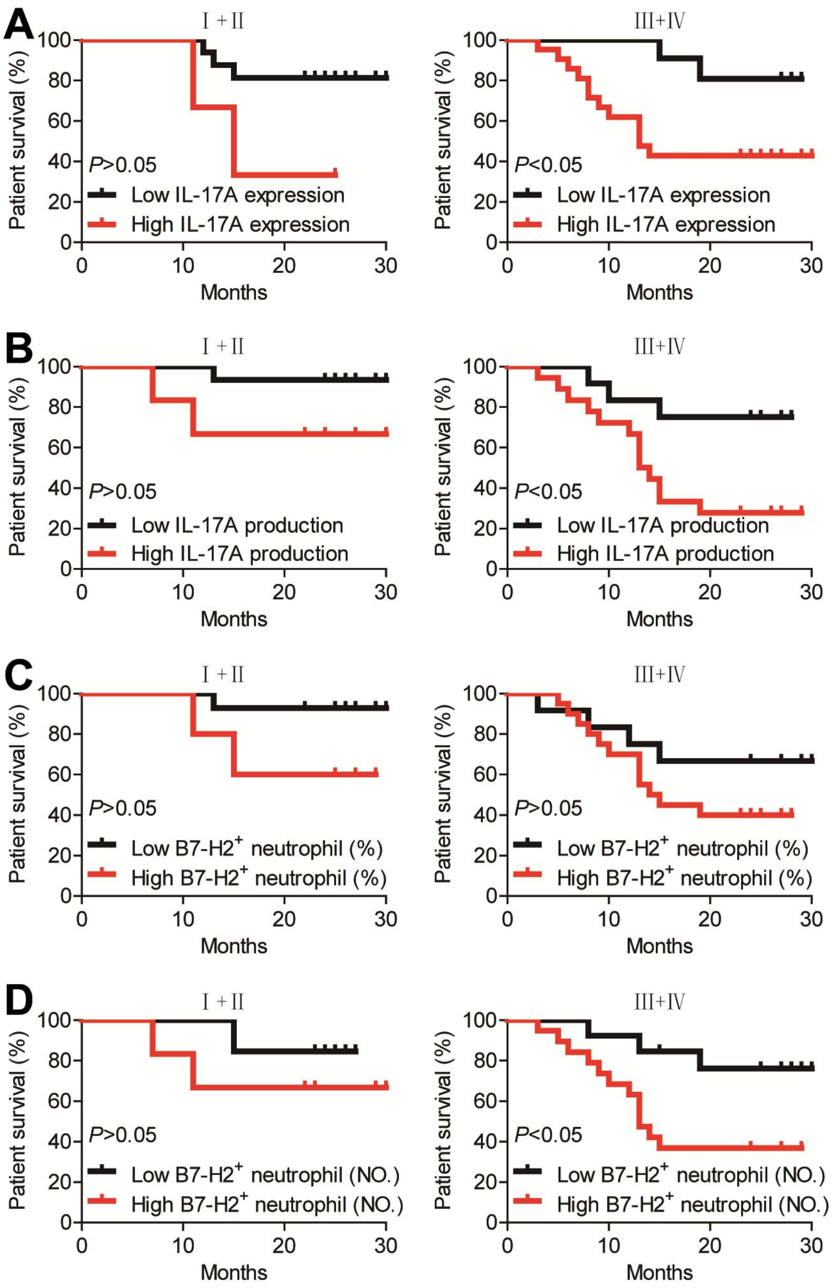

Supplementary Figure 8. B7-H2$^+$ neutrophils and IL-17A correlate with poor survival in patients with GC. (A) Kaplan-Meier plots for overall survival of the GC patients with TNM stage (I+II) or with TNM stage (III+IV) by median IL-17A expression respectively. (B) Kaplan-Meier plots for overall survival of the GC patients with TNM stage (I+II) or with TNM stage (III+IV) by median IL-17A production respectively. (C) Kaplan-Meier plots for overall survival of the GC patients with TNM stage (I+II) or with TNM stage (III+IV) by median B7-H2$^+$ neutrophil percentage respectively. (D) Kaplan-Meier plots for overall survival of the GC patients with TNM stage (I+II) or with TNM stage (III+IV) by median B7-H2$^+$ neutrophil number respectively.

Supplementary Figure 9.

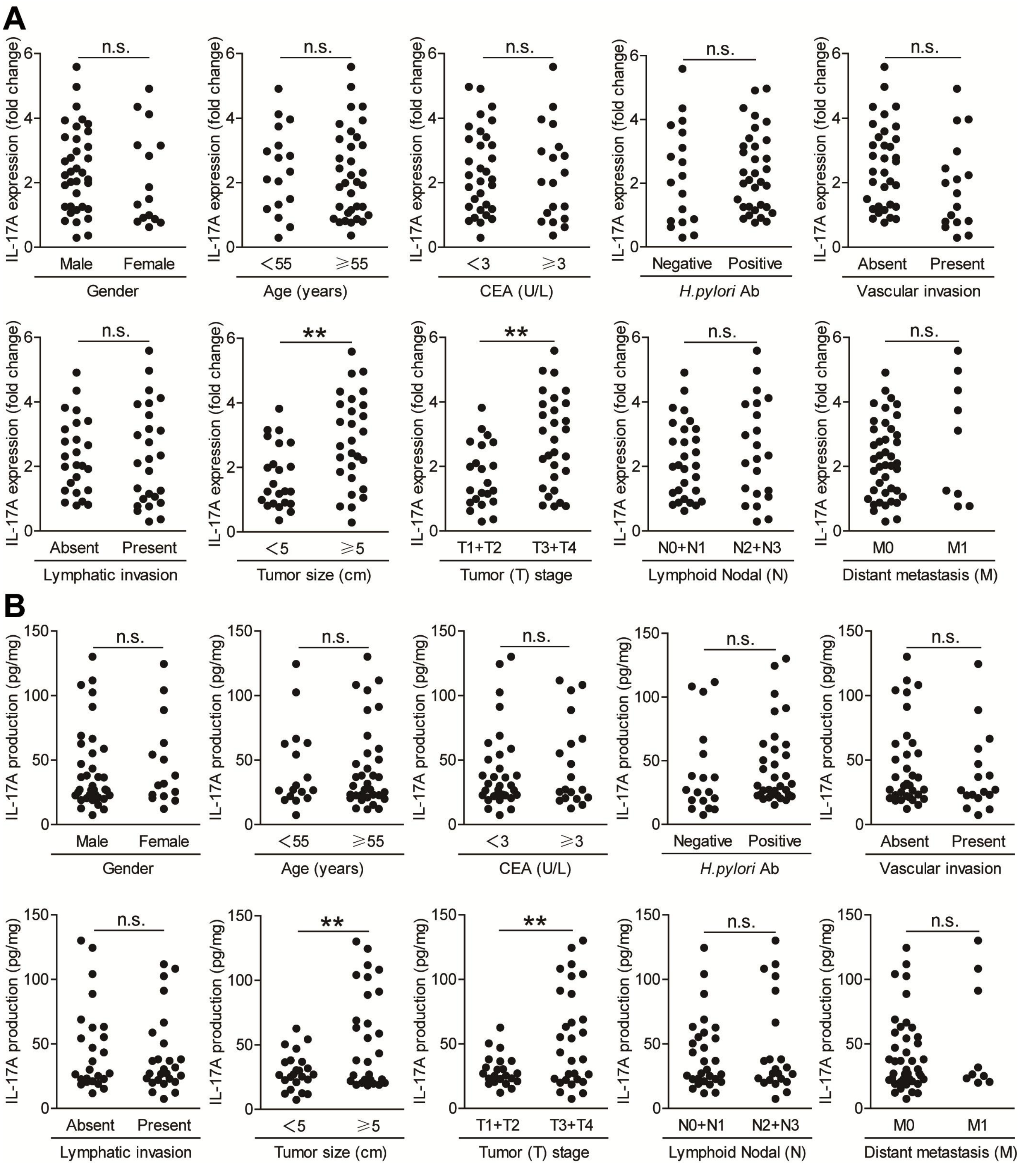

Supplementary Figure 9. IL-17A and its potential correlations with clinical parameters. IL-17A expression (A) or IL-17A production (B) was analyzed for correlations with clinical pathological parameters. **, P<0.01; n.s., P>0.05 for groups connected by horizontal lines. Each dot represents 1 patient. CEA, carcinoembryonic antigen; *H.pylori* Ab, *Helicobacter pylori* antibody.

Supplementary Figure 10.

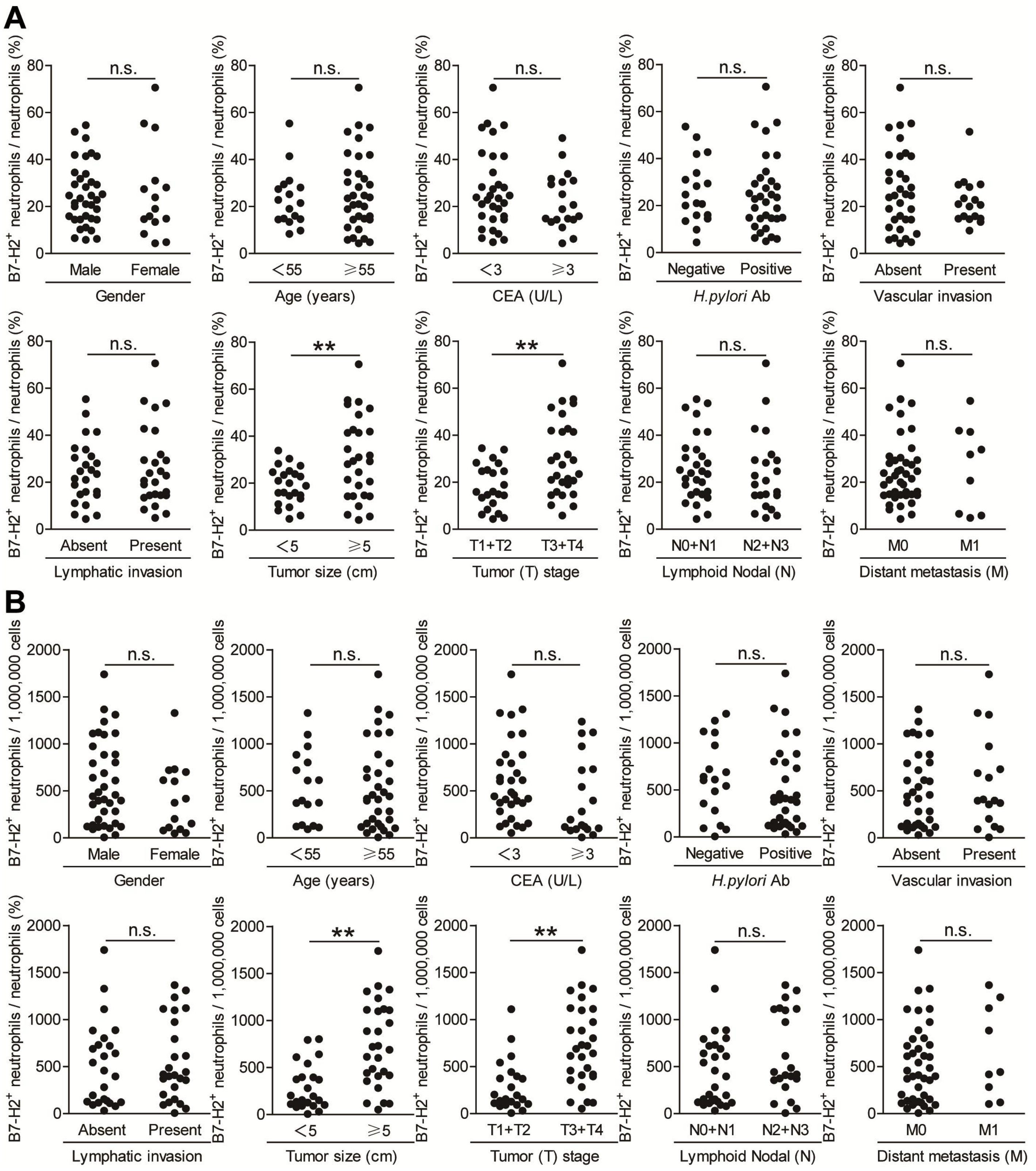

Supplementary Figure 10. B7-H2$^+$ neutrophils and their potential correlations with clinical parameters. B7-H2$^+$ neutrophil percentage (A) or B7-H2$^+$ neutrophil number (B) was analyzed for correlations with clinical pathological parameters. **, *P*<0.01; n.s., *P*>0.05 for groups connected by horizontal lines. Each dot represents 1 patient. CEA, carcinoembryonic antigen; *H.pylori* Ab, *Helicobacter pylori* antibody.
