# Supplementary material for: Activated neutrophils polarize protumorigenic interleukin‐17A‐producing T helper subsets through TNF‐α‐B7‐H2‐dependent pathway in human gastric cancer
Source: Clin Transl Med. 2021 Jun 27;11(6):e484. doi: 10.1002/ctm2.484 (PMC8236123; doi:10.1002/ctm2.484)
Supplement: Supplementary file 2 — Supporting information. [file CTM2-11-e484-s001.doc]

**Supplementary Table 1.** Antibodies and other reagents

| Antibodies and reagents | Manufacturers |
| --- | --- |
| Antibodies for flow cytometry |  |
| anti-CD45-PE-Cy7 | Biolegend |
| anti-CD11b-PerCP-Cy5.5 | Biolegend |
| anti-CD66b-FITC | Biolegend |
| anti-CD15-APC-Cy7 | Biolegend |
| anti-CD54-PE  anti-B7-H2-APC  anti-CXCR1-PE  anti-CD4-Alexa Fluor® 700 | Biolegend  Biolegend  Biolegend  Biolegend |
| anti-IL-17A-PE | Biolegend |
| anti-IL-17A-PE-Cy7  anti-Ki-67-PE  anti-CCR1-PE  anti-CCR2-Brilliant Violet 605™  anti-CCR5-APC | Biolegend  Biolegend  Biolegend  Biolegend  Biolegend |
| anti-CD3-APC-H7 | BD Pharmingen |
| Antibodies for immunohistochemical staining |  |
| rabbit anti-human CD15 | Abcam |
| mouse anti-human CD66b | Biolegend |
| rabbit anti-human IL-17A | Abcam |
| mouse anti-human CD4 | Biolegend |
| anti-human proliferating cell nuclear antigen (PCNA) | Abcam |
| horseradish peroxidase anti-rabbit IgG  horseradish peroxidase anti-mouse IgG  DAB kit  ImmPRESS* Deut Double Staining Polymer Kit (anti-rabbit IgG/HRP+anti-mouse IgG/AP) | Zhongshan Biotechnology  Zhongshan Biotechnology  Zhongshan Biotechnology  Vectorlabs |
| Antibodies for immunofluorescence  rabbit anti-human CD15  mouse anti-human CD4  goat anti-rabbit-FITC  goat anti-mouse-TRITC | Abcam  Abcam  Zhongshan Biotechnology  Zhongshan Biotechnology |
| Antibodies for neutralizing and blocking |  |
| anti-human CXCL6 (Mouse IgG1)  Mouse IgG1 Isotype Control  anti-human CXCL8 (Mouse IgG1)  Mouse IgG1 Isotype Control | R&D Systems  R&D Systems  R&D Systems  R&D Systems |
| anti-human CXCR1 (Mouse IgG2a)  Mouse IgG2a Isotype Control  anti-human IL-17A (Rabbit IgG)  Rabbit IgG Control | Abcam  Abcam  Abcam  Abcam |
| anti-human B7-H2 (Mouse IgG2b)  Mouse IgG2b Isotype Control  anti-human TNF-α (Mouse IgG1)  Mouse IgG1 Isotype Control  anti-human CD54 (Mouse IgG1)  Mouse IgG1 Isotype Control | R&D Systems  R&D Systems  R&D Systems  R&D Systems  R&D Systems  R&D Systems |
| Antibodies for western blot |  |
| anti-human p-p65  anti-human p65 | Abcam  Abcam |
| anti-human ERK1/2 | Abcam |
| anti-human p-ERK1/2 | Abcam |
| anti-human GAPDH | Abcam |
| anti-human Lamin B1 | Abcam |
| Purified anti-CD3 and anti-CD28 antibodies | Biolegend |
| ELISA kits |  |
| TNF-α  CXCL6 | R&D Systems  Abcam |
| CXCL8 | Abcam |
| IL-17A | Abcam |
| IFN-γ | Biolegend |
| IL-4 | Biolegend |
| TGF-β | Biolegend |
| Reagents for signaling pathways inhibition |  |
| MEK-1 and MEK-2 inhibitor U0126 | Merk Millipore |
| IκBα inhibitor BAY 11-7082 | Calbiochem |
| JNK inhibitor SP600125 | Calbiochem |
| MAPK inhibitor SB203580 | Calbiochem |
| PI3K inhibitor Wortmannin  STAT3 phosphorylation inhibitor FLLL32  JAK signaling inhibitor AG490  GSK-3β inhibitor VI | Calbiochem  MedKoo Biosciences  Merk Millipore  Calbiochem |
| CD3 microbeads | Milteniy Biotec |
| CD4 microbeads | StemCell Technologies |
| 3-μm pore size Transwells | Corning |
| Collagenase IV | Gibco |
| DNase I | Sigma-Aldrich |
| Phorbol myristate acetate | Sigma-Aldrich |
| Ionomycin | Sigma-Aldrich |
| DMSO | Sigma-Aldrich |
| Golgistop and Perm/Wash solution | BD Pharmingen |
| Carboxylfluorescein succinimidyl ester (CFSE) | eBioscience |
| Protein Extraction Reagent | Pierce |
| SuperSignal® West Dura Extended Duration Substrate kit | Thermo |
| Fetal calf serum (FCS) | Gibco |
| Penicillin/Streptomycin | Gibco |
| RPMI-1640 | Hyclone |
| Ficoll-Paque Plus | GE Healthcare |
| lyses solution  CCK-8 Kits  TRIzol reagent | TIANGEN  Dojindo  Invitrogen |
| BCA Protein Assay Kit | Abcam |
| PrimeScriptTM RT reagent Kit | TaKaRa |
| Real-time PCR Master Mix | Toyobo |
| All recombinant cytokines and chemokines | PeproTech |

APC-Cy7, allophycocyanin-cyanin 7; PE-Cy7, phycoerythrin-cyanin 7; FITC, Fluorescein isothiocyanate; PE, phycoerythrin; PerCP-Cy5.5, peridin chlorophyl protein-cyanin 5.5; APC, allophycocyanin; IL, interleukin; PCNA, proliferating cell nuclear antigen; TNF-α, tumor necrosis factor-α.

**Supplementary Table 2.** Clinical characteristics of 51 patients with gastric cancer

| Variables | No. of patients |
| --- | --- |
| Gender (male/female) | 36/15 |
| Age (years; median, range) | 61, 24-78 |
| *H.pylori* Ab (negative/positive) | 18/33 |
| CEA (U/L; <5/≥5) | 32/19 |
| Tumor size (cm; <5/≥5) | 23/28 |
| Lymphatic invasion (absent/present) | 25/26 |
| Vascular invasion (absent/present) | 34/17 |
| Tumor (T) invasion (T1+T2/T3+T4) | 22/29 |
| Lymphoid Nodal (N) status (N0+N1/N2+N3) | 29/22 |
| Distant metastasis (M) status (M0/M1) | 42/9 |
| TNM stage (I+II/III+IV) | 19/32 |
| CD15^+^ neutrophil number^a^ (median, range) | 112, 14-364 |
| CD66b^+^ neutrophil number^b^ (median, range) | 111, 13-326 |
| B7-H2^+^ neutrophil percentage^c^ (median, range) | 22.8, 4.37-70.6 |
| B7-H2^+^ neutrophil number^d^ (median, range) | 416, 5-1739 |
| IL-17A expression (fold change) (median, range) | 2.1, 0.29-5.59 |
| IL-17A production (pg/mg) (median, range) | 30.01721, 7.459925-130.1159 |

^a^CD15^+^ neutrophil number was acquired by immunohistochemical staining and counting and was expressed as number per field. ^b^CD66b^+^ neutrophil number was acquired by immunohistochemical staining and counting and was expressed as number per field. ^c^B7-H2^+^ neutrophil percentage was acquired on CD45^+^CD11b^+^CD66b^+^CD15^+^B7-H2^+^ cells that gated on CD45^+^CD11b^+^CD66b^+^CD15^+^ cells of tumor tissues. ^d^B7-H2^+^ neutrophil number was acquired by counting CD45^+^CD11b^+^CD66b^+^CD15^+^B7-H2^+^ cells per million cells of tumor tissues. CEA, carcinoembryonic antigen; *H.pylori* Ab, *Helicobacter pylori* antibody.

**Supplementary Table 3.** Primer and probe sequences for real-time PCR analysis

| Gene | Primer | Sequence 5′→3′ |
| --- | --- | --- |
| Human TNF-α  Human CXCL6  Human CXCL8  Human IL-17A  Human GAPDH | forward  reverse  forward  reverse  forward  reverse  forward  reverse  forward  reverse | TGGCGTGGAGCTGAGAGATAACC  CGATGCGGCTGATGGTGTGG  TCCAAGGTGGAAGTGGTAGC  AGAAAACTGCTCCGCTGAAG  CTGAGAGTGATTGAGAGTGG  ACAACCCTCTGCACCCAGTT  GAGATATCCCTCTGTGATCTGG  GACAGAGTTCATGTGGTAGTCC  ACCCAGAAGACTGTGGATGG  CAGTGAGCTTCCCGTTCAG |

**Supplementary Table 4. Univariate and multivariate analyses of factors associated with overall survival**

| Variables | Univariate | Multivariate | | |
| --- | --- | --- | --- | --- |
|  | *P*-value | HR | 95% CI | *P*-value |
| Gender (male vs. female) | 0.875 |  |  | NA |
| Age, years (≥ 65 vs. < 65) | 0.191 |  |  | NA |
| *H.pylori* Ab (positive vs. negative) | 0.784 |  |  | NA |
| CEA,U/L (≥ 5 vs. < 5) | 0. 224 |  |  | NA |
| Tumor size, cm (≥ 5 vs. < 5) | 0.055 |  |  | NA |
| Lymphatic invasion (positive vs. negative) | 0.447 |  |  | NA |
| Vascular invasion (positive vs. negtive) | 0.857 |  |  | NA |
| Tumor (T) invasion (T1+T2 vs. T3+T4) | 0.075 |  |  | NA |
| Lymphoid Nodal (N) status (N0+N1 vs. N2+N3) | 0.110 |  |  | NA |
| Distant metastasis (M) status (M0 vs. M1) | 0.779 |  |  | NA |
| TNM stage (I+II vs. III+IV) | 0.011 | 7.681 | 1.631-36.177 | 0.010 |
| B7-H2^+^ neutrophil percentage^a^ (high vs. low) | 0.235 |  |  | NA |
| B7-H2^+^ neutrophil number^b^ (high vs. low) | 0.012 | 1.393 | 0.459-4.232 | 0.559 |
| IL-17A expression (fold change) (high vs. low) | 0.011 | 3.238 | 1.125-9.320 | 0.029 |
| IL-17A production (pg/mg) (high vs. low) | 0.006 | 4.223 | 1.306-13.661 | 0.016 |

Cox proportional hazards regression model. Variables used in multivariate analysis were adopted by univariate analysis. ^a^B7-H2^+^ neutrophil percentage was acquired on CD45^+^CD11b^+^CD66b^+^CD15^+^B7-H2^+^ cells that gated on CD45^+^CD11b^+^CD66b^+^CD15^+^ cells of tumor tissues. ^b^B7-H2^+^ neutrophil number was acquired by counting CD45^+^CD11b^+^CD66b^+^CD15^+^B7-H2^+^ cells per million cells of tumor tissues. CEA, carcinoembryonic antigen; *H.pylori* Ab, *Helicobacter pylori* antibody; HR, hazard ratio; CI, confidence interval; NA, not adopted.
